# An Integrative Multiomics Approach to Characterize Prebiotic Inulin Effects on *Faecalibacterium prausnitzii*

**DOI:** 10.3389/fbioe.2022.825399

**Published:** 2022-01-18

**Authors:** Ji-Hyeon Park, Won-Suk Song, Jeongchan Lee, Sung-Hyun Jo, Jae-Seung Lee, Hyo-Jin Jeon, Ji-Eun Kwon, Ye-Rim Kim, Ji-Hyun Baek, Min-Gyu Kim, Yung-Hun Yang, Byung-Gee Kim, Yun-Gon Kim

**Affiliations:** ^1^ Department of Chemical Engineering, Soongsil University, Seoul, South Korea; ^2^ School of Chemical and Biological Engineering, Seoul National University, Seoul, South Korea; ^3^ Department of Biological Engineering, Konkuk University, Seoul, South Korea

**Keywords:** *Faecalibacterium prausnitzii*, inulin, prebiotics, LC-MS/MS, beta-fructosidase, amylosucrase, ABC transporter, PTS transporter

## Abstract

*Faecalibacterium prausnitzii*, a major commensal bacterium in the human gut, is well known for its anti-inflammatory effects, which improve host intestinal health. Although several studies have reported that inulin, a well-known prebiotic, increases the abundance of *F. prausnitzii* in the intestine, the mechanism underlying this effect remains unclear. In this study, we applied liquid chromatography tandem mass spectrometry (LC-MS/MS)-based multiomics approaches to identify biological and enzymatic mechanisms of *F. prausnitzii* involved in the selective digestion of inulin. First, to determine the preference for dietary carbohydrates, we compared the growth of *F. prausnitzii* in several carbon sources and observed selective growth in inulin. In addition, an LC-MS/MS-based intracellular proteomic and metabolic profiling was performed to determine the quantitative changes in specific proteins and metabolites of *F. prausnitzii* when grown on inulin. Interestingly, proteomic analysis revealed that the putative proteins involved in inulin-type fructan utilization by *F. prausnitzii*, particularly β-fructosidase and amylosucrase were upregulated in the presence of inulin. To investigate the function of these proteins, we overexpressed *bfrA* and *ams,* genes encoding β-fructosidase and amylosucrase, respectively, in *Escherichia coli,* and observed their ability to degrade fructan. In addition, the enzyme activity assay demonstrated that intracellular fructan hydrolases degrade the inulin-type fructans taken up by fructan ATP-binding cassette transporters. Furthermore, we showed that the fructose uptake activity of *F. prausnitzii* was enhanced by the fructose phosphotransferase system transporter when inulin was used as a carbon source. Intracellular metabolomic analysis indicated that *F. prausnitzii* could use fructose, the product of inulin-type fructan degradation, as an energy source for inulin utilization. Taken together, this study provided molecular insights regarding the metabolism of *F. prauznitzii* for inulin, which stimulates the growth and activity of the beneficial bacterium in the intestine.

## Introduction

In the human gastrointestinal tract (GI), there is a complex and dense microbial community that maintains a symbiotic relationship with the host (Bäckhed et al., 2005; Eckburg et al., 2005; El Kaoutari et al., 2013). It degrades monosaccharides and dietary carbohydrates, which are resistant to human digestive enzymes and ferments them into short-chain fatty acids (SCFAs) (Flint et al., 2012; Nyangale et al., 2012). Among the microbial-derived SCFAs, butyrate acts as an energy source for colonocytes and plays an important role in regulating intestinal health and host immune system by providing anti-carcinogenic, anti-inflammatory, and barrier protection in the intestine (Roediger, 1980; Hamer et al., 2008; Fung et al., 2012; Wang et al., 2012). Recently, based on the correlation between host health and fermentation metabolites produced by beneficial gut bacteria, there is a growing interest in intentionally increasing the proportion of butyrate-producing bacteria in the gut.

*Faecalibacterium prausnitzii*, a member of Clostridium cluster IV in Bacillota (previously known as Firmicutes), is one of the major butyrate-producing bacteria in the intestine (Miquel et al., 2013; Oren and Garrity, 2021). It has recently emerged as a potential next-generation probiotic that plays an important role in regulating intestinal inflammatory responses and maintaining host gut health (Walker et al., 2011; O’Toole et al., 2017). *F. prausnitzii* is one of the most abundant microbial species, accounting for approximately 5–15% of the human gut microbiota and is less abundant in the intestines of individuals suffering from irritable bowel syndrome, obesity, type 2 diabetes, and inflammatory bowel diseases such as ulcerative colitis, and Crohn’s disease (Jia et al., 2010; Willing et al., 2010; Joossens et al., 2011; Miquel et al., 2013; Miquel et al., 2014; Lopez-Siles et al., 2016). In addition, regulating the intestinal proportion of *F. prausnitzii* is expected to alleviate or treat these diseases. The bacterium has been reported to reduce the severity of intestinal inflammation through anti-inflammatory metabolites in a colitis mouse model (Sokol et al., 2008; Martín et al., 2015; Miquel et al., 2015). However, several constraints need to be addressed prior to commercialization, such as the need to minimize contact with external oxygen since *F. prausnitzii*, an obligate anaerobe, loses viability within 2 min of exposure to ambient air (Duncan et al., 2002; Khan et al., 2014). Therefore, to increase the abundance and activity of *F. prausnitzii* in the intestine, a dietary carbohydrate that can selectively stimulate the metabolic activity and proliferation of *F. prausnitzii* is required.

Prebiotics are nutrients that are not digested by the human gastrointestinal tract, but can improve human health by selectively stimulating the growth and activity of the gut microbiota (Gibson and Roberfroid, 1995). Inulin is one of the most widely used prebiotics as a component of food and nutritional supplements. It consists of β-2,1-linked fructosyl residues with an α-1,2-linked glucose terminus and has a linear structure with a degree of polymerization (DP) of 2–60 (Roberfroid and Delzenne, 1998; Roberfroid, 2005). An *in vitro* study has demonstrated the degradation of inulin by *F. prausnitzii*, and ingestion of a diet containing inulin-type fructan increased the abundance of *F. prausnitzii* in the human gut (Duncan et al., 2002; Ramirez-Farias et al., 2008; Dewulf et al., 2013; Moens and De Vuyst, 2017). Furthermore, increased butyrate production by consuming an inulin-rich diet has been observed in a mouse model, and increasing evidence support the effect of inulin on intestinal health in various *in vivo* models (Campbell et al., 1997; Koleva et al., 2012; Holscher et al., 2014). However, despite considerable interest in the role of *F. prausnitzii* as an efficient degrader of inulin and the subsequent effect on improving intestinal health, molecular-level studies to determine the mechanisms whereby *F. prausnitzii* utilizes dietary inulin and undergoes physiological changes are still insufficient.

In this study, integrative multiomics analysis was performed on proteins and metabolites, to identify the biological mechanism involved in the selective digestion of inulin in the intestine by *F. prausnitzii*. To investigate molecular-level cellular metabolic changes using multiomics approaches, we used *F. prausnitzii* strain A2-165, whose complete genome sequence has already been well reported (Miquel et al., 2013; Fitzgerald et al., 2018; Ueda et al., 2021). First, to confirm the preference for dietary carbohydrates in *F. prausnitzii*, we compared the growth of the bacterium in the presence of several carbon sources and confirmed significant growth in inulin. In addition, liquid chromatography with tandem mass spectrometry (LC-MS/MS)-based intracellular proteome analysis was performed to determine quantitative changes in the protein content of *F. prausnitzii* during growth on inulin as the sole carbon source. The results showed that the proteins involved in inulin degradation and intracellular transport were upregulated, and fructan hydrolysis enzymes, β-fructosidase and amylosucrase, were significantly upregulated. Expression levels of the genes encoding these two enzymes were determined using quantitative real time polymerase chain reaction (qRT-PCR). The activity of the enzymes against inulin and sucrose degradation was demonstrated by overexpression in *Escherichia coli*. Analysis of enzyme activity and subcellular location showed that fructan hydrolases are cytoplasmic enzymes that degrade the inulin-type fructan taken up by the fructan ATP-binding cassette (ABC) transporters, which are upregulated in proteomic data. In addition, upregulation of the fructose phosphotransferase system (PTS) transporter, which transports fructose into cells, indicates that *F. prausnitzii* can also enhance the uptake fructose through inulin metabolism. Furthermore, intracellular metabolome analysis demonstrated that *F. prausnitzii* used fructose, the final degradation product of inulin-type fructan, as an efficient energy source during the inulin utilization process. In this study, we characterized the functional roles of proteins upregulated in inulin metabolism by *F. prausnitzii* on a molecular scale; based on these data, we were able to outline a model in which *F. prausnitzii* utilizes inulin in the intestinal ecosystem. Finally, understanding these metabolic pathways is expected to provide evidence for the development of targeted prebiotics for enhancing intestinal microorganisms classified as beneficial bacteria.

## Materials and Methods

### Bacterial Strain and Culture Condition

The bacterial strains used in this study were *F. prausnitzii* A2-165 (DSM 17677), *Eubacterium rectale* (KCTC 5835), *Roseburia hominis* (KCTC 5845), and *Roseburia intestinalis* (KCTC 15746). *F. prausnitzii* A2-165 (DSM 17677) was purchased from German collection of microorganisms and cell cultures (DSMZ, Braunschweig, Germany) and cultured in modified YCFA medium, hereafter referred to as mYCFA medium. All components were added as the referenced method, but the final concentration of short-chain fatty acids (SCFA) in the medium was modified (Duncan et al., 2002). 30 mM of acetic acid, sterilized by syringe filter (PVDF, pore size 0.45 μm, JETbiofil), was added to the sterilized medium. If 5 g/L glucose (Sigma-Aldrich, MO, United States) was supplemented as a carbon source, hereafter referred to as mYCFAG.

*F. prausnitzii* was cultured on mYCFA agar and a preculture was prepared by inoculating single colony of *F. prausnitzii* in 5 ml of mYCFAG broth. Precultured *F. prausnitzii* was diluted in 30 ml of fresh mYCFAG broth to an initial optical density of 0.05 at 600 nm and cultured in 37°C anaerobic chamber (Coy Laboratory Products, MI, United States) containing 86% N_2._, 7% CO_2_ and 7% H_2_. Optical densities were measured by an UV spectrophotometry (Thermo Fisher Scientific, MA, United States). To culture *F. prausnitzii* in mYCFA containing a carbohydrate source, 300 μL in the mid-log phase cultured in mYCFAG medium was inoculated in the 30 ml of mYCFA containing a carbohydrate source at 0.3% (w/v) final concentration. Glucose (Sigma-Aldrich, MO, United States), inulin (Megazyme, IL, United States), amylopectin (Sigma-Aldrich, MO, United States), arabinan (Megazyme, IL, United States), arabinoxylan (Sigma-Aldrich, MO, United States), xylan (Sigma-Aldrich, MO, United States), galactan (Sigma-Aldrich, MO, United States), lichenan (Sigma-Aldrich, MO, United States), and pullulan (Sigma-Aldrich, MO, United States) were used as sole carbon sources, respectively. These were sterilized through membrane filtration using syringe filter (CA, pore size 0.2 μm, Hyundai Micro), then added to the sterilized mYCFA medium.

### SCFAs Analysis by LC-MS/MS

For the SCFAs analysis, we followed the referenced method with some modifications (Song et al., 2020). The culture supernatants of *F. prausnitzii* were harvested by centrifugation at each time points and filtrated with a syringe filter (PVDF, pore size 0.45 μm, Millipore). Then 50 μL of filtered supernatant was mixed with 450 μL HPLC-grade water (Fisher Scientific, NH, United States). Butyrate-d7 (CDN isotopes, QC, Canada) was used as internal standard compound, and dissolved in 50% Acetonitrile (ACN, Fisher Scientific, NH, United States) to 1 mM. Girard’s reagent T (GT, Sigma-Aldrich, MO, United States), used for chemical derivatization, was dissolved in 50% ACN with 40 μL/ml pyridine (Sigma-Aldrich, MO, United States) and 18 μL/ml HCl (Junsei, Tokyo, Japan) to 100 mM. Also 100 mM 1-ethyl-3-(3-dimethylaminopropyl)carbodiimide (EDC, Sigma-Aldrich, MO, United States) were dissolved in 50% ACN. GT derivatization reaction conditions were prepared as follows. First, 20 μL of diluted supernatant was mixed with 10 μL of butyrate-d7, 40 μL of 100 mM GT, 40 μL of 100 mM EDC, and 50 μL 50% of ACN followed by incubation at 40°C for 1 h. After incubation, it was diluted 100-fold with 50% ACN and analyzed by LC–MS/MS. To analyze the quantitative curve of GT-labeled butyrate, the SCFA standard mixture consists of acetate, propionate, butyrate, valerate, caproate (Sigma-Aldrich, MO, United States) was dissolved in 50% ACN (100 nmol/ml). It was serially diluted, and 20 μL of the diluted SCFA standard mixture was GT derivatizated and incubated as above. After incubation, it was diluted 20-fold with 50% ACN and analyzed by LC–MS/MS. The number of molecules of GT-labeled SCFA injected into LC–MS/MS was as follows: 1,000 pmol, 200 pmol, 50 pmol, 10 pmol, 2 pmol, 500 fmol, 100 fmol, 20 fmol, 5 fmol, 1 fmol, and 0 fmol.

LC–MS/MS analysis was performed using an integrated system composed of Acquity UPLC H-Class (Waters, MA, United States) and LTQ XL™ Linear Ion Trap Mass Spectrometer (Thermo Fisher Scientific, MA, United States). Then 5 μL of reaction mixture of GT-labeled butyrate was injected into Zorbax HILIC plus column (4.6 × 100 mm, 3.5 μm, Agilent, CA, United States). Solvent C comprised water containing 20 mM ammonium acetate and 20 mM acetic acid while solvent D was 100% ACN. GT-labeled SCFAs were separated on the analytical column at a flow rate of 0.3 ml/min. The LC gradient method was set as follows: t = 0 min, 70% D; 1 min, 70% D; 10 min, 30% D; 15 min, 30% D; 15.1 min, 70% D; and 20 min, 70% D. The mass spectrometer was operated in positive ion mode. The normalized collision energy was 35.

### Preparation of Intracellular Proteomic Analysis

For the preparation of intracellular proteomic analysis, we used the filter-aided sample preparation (FASP) method with some modifications (Wiśniewski et al., 2009). Cell pellets of *F. prausnitzii* were harvested by centrifugation at the mid-log phase and were washed two times with phosphate-buffered saline (PBS) at 4°C. Cell pellets were resuspended with RIPA buffer (Thermo Fisher Scientific, MA, United States) supplemented with 0.1% protease inhibitor cocktail (Sigma-Aldrich, MO, United States) and sonicated on ice by a probe sonicator (Sonics & Materials, Inc., CT, United States) for cell lysis. After cell lysates were centrifuged at 4,000 rpm for 10 min at 4°C, supernatants were obtained. The protein concentration was measured by the bicinchoninic acid protein assay kit (BCA assay; Thermo Fisher Scientific, MA, United States) according to the manufacturer’s instructions. For protein reduction, dithiothreitol (DTT) was added to a final concentration of 50 mM and incubated for 5 min at 95°C. 200 μg of reduced proteins were transferred to 30 k filter units (Microcon; Millipore, MA, United States). 200 μL of UA buffer (8 M urea in 0.1 M Tris-HCl, pH 8.5) was added to the device and centrifuged at 10,000 xg for 15 min at 20°C. This step was repeated 3 times, and proteins were alkylated by addition of 100 μL of 0.05 M iodoacetamide (IAM) in UA buffer and incubation in the dark and at room temperature for 20 min. After centrifugation, the concentrate was diluted with 100 μL of UA buffer and centrifuged and repeated this step twice. Subsequently, 100 μL 0.05 M Tris-HCl, pH 8.5 were added to filter unit and centrifuged again. This step was repeated 2 times, and the prepared proteins were digested by trypsin. To collect the digests, 250 μL of 0.05 M Tris-HCl, pH 8.5 was added to filter unit and centrifuged. Then the digests were desalted on Pierce Peptide Desalting Spin Columns (Thermo Fisher Scientific, MA, United States) according to the instructions provided by the manufacturer. Purified samples were dried with a centrifugal vacuum concentrator (Vision Scientific, Seoul, Korea) and stored at −80°C until used for analysis.

### Intracellular Proteome Analysis by LC-MS/MS

Proteomic analysis was performed as described previously (Jo et al., 2020). Briefly, the dried samples were dissolved in solvent A (0.1% v/v formic acid in water) before the analysis. Proteomic analysis was performed using nano-HPLC, Ultimate 3,000 RSLCnano LC system (Thermo Scientific, MA, United States). Q Exactive Hybrid Quadrupole-Orbitrap (Thermo Scientific, MA, United States) equipped with a nano-electrospray ionization source was used in combination with the nano-HPLC. Samples were trapped in an Acclaim PepMap 100 trap column (100 μm × 2 cm, nanoViper C18, 5 μm, 100 Å, Thermo Scientific, MA, United States). Then, solvent A (98%) was used to wash the column at a flow rate of 4 μL/min for 6 min. After washing, the samples were separated at a flow rate 350 nL/min. An Acclaim PepMap 100 capillary column (75 μm × 15 cm, nanoViper C18, 3 μm, 100 Å, Thermo Scientific, MA, United States) was used for LC separation. The LC gradient was as follows: 0 min, 2% B; 30 min, 35% B; 40 min 90% B, 45 min, 90% B; 60 min, 5% B. Solvent A (0.1% formic acid in water) and solvent B (0.1% formic acid in acetonitrile) were used. The ion spray voltage was 2,100 eV. MS data were collected using Xcaliber software. The Orbitrap analyzer scanned precursor ions with a mass range of 350–1800 m/z and a resolution of 70,000 at m/z 200. For collision-induced dissociation (CID), up to the 15 most abundant precursor ions were selected. The normalized collision energy was 32.

Protein identification and label-free quantification (LFQ) were performed using MaxQuant software (Cox et al., 2014). Proteins were identified by searching MS and MS/MS data of peptides against the UniProt database. Statistical analysis of LFQ data was processed with the R statistical programming environment (Shah et al., 2020). Quantified protein data were processed with Perseus software, and the significance was determined using the adjusted *p*-value (Tyanova et al., 2016).

### Intracellular Metabolite Extraction

A cell pellet was obtained from 10 ml of culture by centrifugation at 3,000 rpm at 4°C for 10 min and washed twice with the sterile PBS at 4°C. Then, 1 ml of 80% cold methanol (−80°C, HPLC grade, Fisher Scientific, NJ, United States) was added to each cell pellet to extract intracellular metabolites. After samples were vortexed for 1 min, they were incubated at −80°C for 1 h. The supernatant was obtained after centrifugation at 13,000 rpm for 3 min at 4°C. For re-extraction, cold 80% methanol was added to cell pellets remained and they were incubated at −80°C for 1 h. Next, supernatant was obtained as described above, and the extraction step of 1 h was repeated one more. Finally, the metabolite samples were dried using centrifugal vacuum concentrator (Hanil Science Industrial, Gimpo, Korea) and stored at −80°C before analysis.

### Intracellular Metabolome Analysis by LC-MS/MS

The metabolite samples were dissolved in 50% methanol containing 10 μg/mL L-Phenylalanine ^13^C_9_, ^15^N (Sigma-Aldrich, MO, United States) as an internal standard (IS). They were filtrated with a syringe filter (PVDF, pore size 0.45 μm, Millipore). Prepared samples were transferred to LC vials, and LC-MS/MS analysis was performed. Analysis was performed using a 1,260 Infinity Binary HPLC system (Agilent, CA, United States) combined with a 6,420 Triple Quadrupole LC-MS system (Agilent, CA, United States). Injected metabolites were separated using an Xbridge amide column (4.6 × 100 mm, 3.5 μm particle size, Waters, MA, United States) with solvent A (25 mM ammonium acetate (HPLC grade, Fisher Scientific, NJ, United States) and 25 mM ammonium hydroxide (HPLC grade, Fisher Scientific, NJ, United States) in 95: 5 = water: ACN) and solvent B (100% ACN) given a gradient to analysis. The flow rate was 0.4 ml/min and the electrospray ionization voltage was 4 kV. The LC gradient was as follows: 0 min, 85% B; 5 min, 45% B; 16 min, 0% B; 24 min, 0% B; 25 min, 85% B; 32 min, 85% B. MS peak areas were extracted using Agilent MassHunter Qualitative Analysis software. The peak area was normalized using the peak area of internal standard. The False Discovery Rate (FDR)-corrected *p*-values were provided using the Statistical analysis module of Metaboanalyst 5.0 (Pang et al., 2021).

### mRNA Isolation and Quantitative Analysis by qRT-PCR

Cell pellets *of F. prausnitzii* in the mid-log phase were harvested by centrifugation at 4°C and total RNA was isolated by using TRIzol reagent (Takara, Tokyo, Japan), following the supplier’s instructions. The RNA concentration and the purity were measured by spectrophotometry (Thermo Fisher Scientific, MA, United States). The extracted RNA was reverse transcribed into cDNA using a cDNA synthesis kit (Bio-Rad, CA, United States). PCR primer sequences for β-fructosidase, amylosucrase, and GAPDH were designed in this study by using SnapGene software (Version 3.1; GSL Bio-tech, snapgene.com) and shown in Supplementary Table S6. GAPDH was used as an internal control for normalize the mRNA expression levels of all genes. The sample mixtures for qRT-PCR were prepared with SsoAdvanced Universal SYBR Green Supermix (Bio-Rad, CA, United States). The qRT-PCR was performed using the CFX connect (Bio-Rad, CA, United States) under the following conditions: 40 cycles of denaturation at 95°C for 15 s, annealing at 60°C for 30 s. The results were analyzed using CFX Maestro software (Bio-Rad, CA, United States). All experiments were performed in three biological replicates for each experiment.

### Cloning of *bfrA* Gene and *ams* Gene

The β-fructosidase gene was amplified from genomic DNA of *F. prausnitzii* using primer *bfrA*_F (5′-ATA​TGG​ATC​CAT​GAA​CGA​TTT​GAC​TTT​ACA-3′) and *bfrA*_R (5′-ATA​TCT​CGA​GTT​ACA​GAT​TCA​GTT​CAA​ACT-3′). And the amylosucrase gene was amplified from genomic DNA of *F. prausnitzii* using *ams*_F (5′-ATA​TGG​ATC​CAT​GGC​AGC​AAA​ACA​GGA​ATT-3′) and *ams*_R (5′-ATA​TCT​CGA​GTT​ATT​TGT​TTT​TTA​CCA​GCA-3′). The DNA fragments were amplified by PCR with an Automated Thermal Cycler (Thermo Fisher Scientific, MA, United States) from the following PCR mixture: 50 ng genomic DNA, 1 μM of each primer, 0.2 mM of each dNTP, polymerase buffer, and herculase II Fusion DNA Polymerase (Agilent, CA, United States). PCR conditions were 95°C for 5 min, followed by 25 cycles of 95°C for 30 s, 50°C for 30 s, and 72°C for 1 min, with a final extension period of 5 min at 72°C. The PCR products were purified and cloned into the pET28a, yielding pET28a-*bfrA* and pET28a-*ams*. The cloning products were heat-transformed into *E. coli* strain DH5α. After the colony selection on LB agar plate with ampicillin, the selected colony was grown overnight in LB broth at 37°C. Constructed vectors were extracted and stored at −70°C.

### Protein Expression

β-fructosidase and amylosucrase were expressed using *E. coli* BL21 (DE3). The constructed vectors were transformed into competent cells of *E. coli* BL21. *E. coli* BL21 cells containing pET28a-*bfrA* and pET28a-*ams* were cultured at 37°C in LB medium containing 100 μg/ml ampicillin until OD600 nm reached 0.6–0.8. Expressions of *bfrA* and *ams* were induced by addition of 0.5 mM isopropyl-β-d-thiogalactoside (IPTG) with subsequent overnight incubation at 18°C. The cells were harvested by centrifugation (3,500 xg, 10 min, 4°C), and the pellet was resuspended in Tris buffer solution containing 50 mM Tris-HCl (pH 8.0), 200 mM NaCl and 10% (v/v) glycerol. After ultrasonification in ice-chilled water, the extracts were centrifuged (13,000 xg, 30 min, 4°C) and the supernatants were harvested. To confirm the protein molecular weight, the soluble fraction of lysates was loaded into 12% sodium dodecyl sulfate-polyacrylamide gel electrophoresis (SDS–PAGE) at 50 V for 15 min and 130 V for 60 min after the denaturation process (Supplementary Figure S1). Protein concentrations were measured using the Bradford assay.

### Enzymatic Function Assay

The enzymatic functions were measured in a 50 µL volume that contained 36 ng of proteins in soluble fractions of the cell lysates and 10 mg/ml of sucrose or inulin in 50 mM sodium phosphate buffer. The reaction was performed at 37°C for 10 min and stopped by heating at 95°C for 10 min. The reaction products were centrifuged (16,000 xg, 5 min, 4°C) and mass spectra of the supernatant were analyzed by matrix-assisted laser desorption ionization-time of flight mass spectrometry (MALDI-TOF-MS, Bruker Daltonics, MA, United States). Briefly, 1 μL of the supernatants was mixed with 1 μL of 2,5-dihydroxybenzoic acid (DHB) solution. And 1 μL of the mixture was spotted and dried on a stainless steel MALDI plate. MALDI spectra was obtained by scanning a total of 600 shots from six different spots in positive ion mode. The operating conditions were as follows: accelerating voltage 20 kV; laser frequency 60 Hz; ion source 1 voltage 19 kV; ion source 2 voltage 16 kV; lens voltage 9.8 kV; detector gain 5.8 and laser power 70%. The average spectrum profiles obtained were visualized with FlexAnalysis 3.3 software (Bruker Daltonics, MA, United States).

### Fructan Hydrolysis Assay

Fructan hydrolysis assay was performed following the referenced methods with some modifications (Goh et al., 2007; Fang et al., 2021). After the cell pellet of *F. prausnitzii* cultured in the mYCFAG was washed and re-suspended in carbohydrate-free mYCFA, 300 μL was inoculated in the 30 ml of mYCFA medium supplemented with 0.3% (w/v) inulin. Cell fractionation was performed when the *F. prausnitzii* was in the mid-log phase. The cells were harvested by centrifugation at 3,000 xg for 15 min at 4°C. The culture supernatant was filtered and sterilized with a syringe filter (PES, pore size 0.2 μm, Hyundai Micro), and concentrated using an Amicon Ultra-4 ultrafiltration centrifuge tube (30 k; Millipore, MA, United States). The cell pellet was washed twice in 100 mM potassium phosphate buffer (pH 7.0) and resuspended in 1 ml of the same buffer. The cell suspension was transferred into microtube and sonicated on ice by a probe sonicator (Sonics & Materials, Inc., CT, United States). Then, the cell lysate was transferred into a new tube, and the fraction containing cell wall fragments was separated from the cytoplasmic fraction by centrifuging at 13,800 xg for 10 min at room temperature. Then the cell wall fraction was resuspended in 1 ml of phosphate buffer, and the cytoplasmic extract was concentrated by using an Amicon Ultra-4 ultrafiltration centrifuge tube.

The concentrated culture supernatant, cell wall fraction, or cytoplasmic extract was added to 190 μL of 0.3% (w/v) sucrose or inulin solution. The reaction mixture was incubated at 37°C for 3 h, and inactivated at 95°C for 3 min. The fructan hydrolysis activity was measured by determining the amount of fructose released per min per mg of protein. The fructose concentration was determined by high-performance liquid chromatography using an Xbridge amide column (Waters, MA, United States). The protein concentration was measured by the BCA assay kit according to the manufacturer’s instructions.

### Fermentation Experiments

For the comparison of the growth of *F. prausnitzii* according to the sole carbon source, the cell pellet of *F. prausnitzii* cultured in the mYCFAG was washed twice with carbohydrate-free mYCFA and re-suspended in carbohydrate-free mYCFA. 300 µL of this suspension was inoculated onto 30 ml of mYCFA containing 0.3% (w/v) glucose, fructose, sucrose, and inulin as the sole carbon source, respectively. Inulin was sterilized at 121°C for 15 min and the others were sterilized through membrane filtration using CA syringe filter, then they were added to the sterilized mYCFA medium. Glucose, fructose was purchased from Sigma-Aldrich (MO, United States), sucrose was purchased from Junsei (Tokyo, Japan), and inulin was purchased from Megazyme (IL, United States). According to the information supplied by the company, the DP of the inulin chains is varied between 2 and 60. *F. prausnitzii* was cultured anaerobically at 37°C and optical densities of the cultures were measured at 600 nm.

Also, the bacterial pellet cultured in mYCFA supplemented with 0.3% (w/v) glucose or inulin was re-suspended in carbohydrate-free mYCFA when the OD600 nm reached 1.11 or 0.57, respectively. Then, 300 µL of this suspension was inoculated onto 30 ml of mYCFA containing 0.3% (w/v) fructose or sucrose. Culture was performed anaerobically at 37°C and optical densities at 600 nm were measured.

### Comparative Genomic Analysis

A BLAST search using the UniProt database was applied to determine the protein sequence alignments and the BLAST hits with a cut-off of E-value <0.0001. The output of sequences producing significant alignments (E-value <0.0001) against other *F. prausnitzii* strains was determined by NCBI BLASTP online tool.

### Statistical Analysis

Statistical analysis was performed using R statistical programming environment. Data was expressed as the means ± s.d. All data was normalized with mean of control, and significant differences of between two groups was assessed to the unpaired two-tailed Student’s *t* test. *p* < 0.05 were defined as quantitatively significant. Statistical comparison was indicated with *, **, ***, **** for *p* < 0.05, *p* < 0.01, *p* < 0.001, *p* < 0.0001 respectively.

## Results and Discussion

### Increased Fermentation Ability and Butyrate Productivity of *F. prausnitzii* Grown on Inulin

**FIGURE 1:**
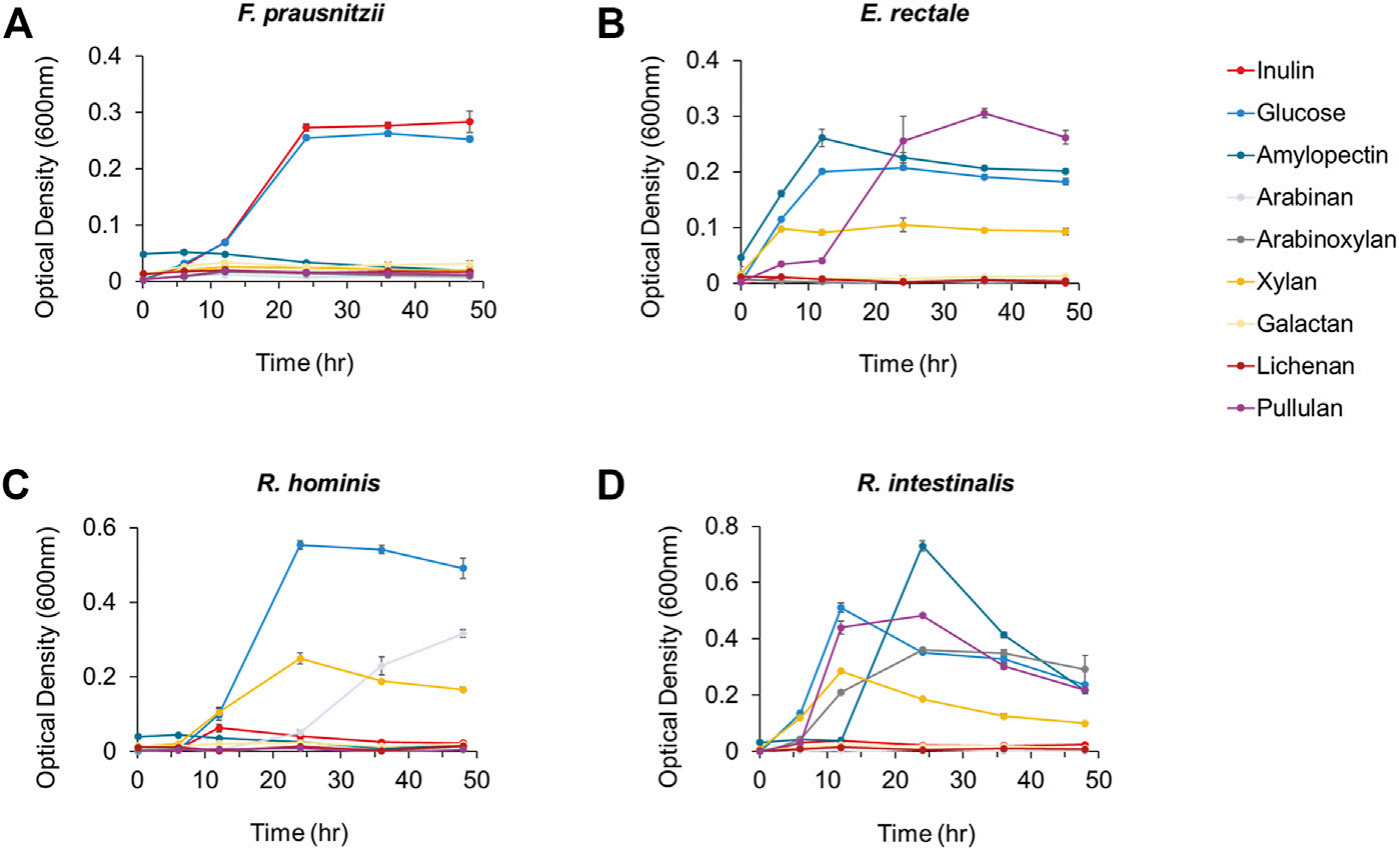
The growth analysis of butyrate-producing bacteria **(A)** *F. prausnitzii* **(B)** *E. rectale* **(C)** *R. hominis* and **(D)** *R. intestinalis* cultured in mYCFA supplemented with either inulin, glucose, amylopectin, arabinan, arabinoxylan, xylan, galactan, lichenan, or pullulan. All experiments were performed in triplicates for each experiment.

**FIGURE 2:**
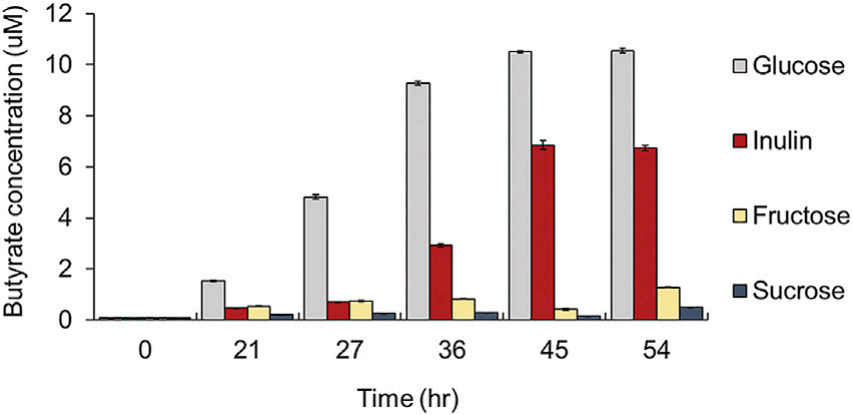
Comparison of butyrate productivity of *F. prausnitzii* grown in mYCFA supplemented with either glucose, inulin, fructose, or sucrose. All experiments were performed in triplicates for each experiment.

First, we added glucose and eight polysaccharides (i.e., inulin, amylopectin, arabinan, arabinoxylan, xylan, galactan, lichenan, and pullulan) as the sole carbon source to a carbohydrate-free medium and observed the growth of four major compositional species among human intestinal butyrate-producing bacteria (i.e., *F. prausnitzii, E. rectale, R. hominis,* and *R. intestinalis*), to explore the preference for dietary fibers*.* Among these butyrate-producing bacteria, only *F. prausnitzii* could grow on inulin as the sole carbon source. While the growth of all strains was enhanced when grown on glucose, *F. prausnitzii* was also able to grow on glucose and inulin, as previously reported (Figure 1) (Duncan et al., 2002; Desai et al., 2016; Louis and Flint, 2017). We also measured the production of butyrate, a beneficial metabolite to the intestine, from *F. prausnitzii* grown on each carbon source; high butyrate productivity was observed when glucose or inulin was used as the sole carbon source (Figure 2). The highest productivity of butyrate was observed after culturing for 45 h on inulin, which was 65% of that produced by using glucose as the carbon source.

To efficiently produce butyrate by using a substrate in a non-competitive manner with other bacteria in the intestine, it was preferable to use highly selective inulin, instead of glucose, as the carbon source for *F. prausnitzii*, which has excellent growth ability against most of the other gut bacteria. Findings from *in vivo* inflammatory models have proved that the ingestion of prebiotics, such as inulin, is effective in promoting host health, by inducing selective growth of butyrate-producing *F. prausnitzii* and possessing immunomodulatory properties, such as intestinal barrier protection and anti-inflammatory effects in (Sokol et al., 2008; Varela et al., 2013; Martín et al., 2015; Miquel et al., 2015; Lopez-Siles et al., 2016). However, research on the precise mechanism and degrading enzymes involved in the selective utilization of inulin in *F. prausnitzii* remains limited; molecular-based characterization is required to induce butyrate production *in vivo* using *F. prausnitzii* and for the commercial use of related strains and enzymes in future. Therefore, we applied integrative proteomics-metabolomics approaches, to explore the changes occurring at the molecular level in inulin utilization by *F. prausnitzii* and to characterize the related degrading enzymes.

### Proteomic Changes of *F. prausnitzii* Grown on Inulin

**FIGURE 3:**
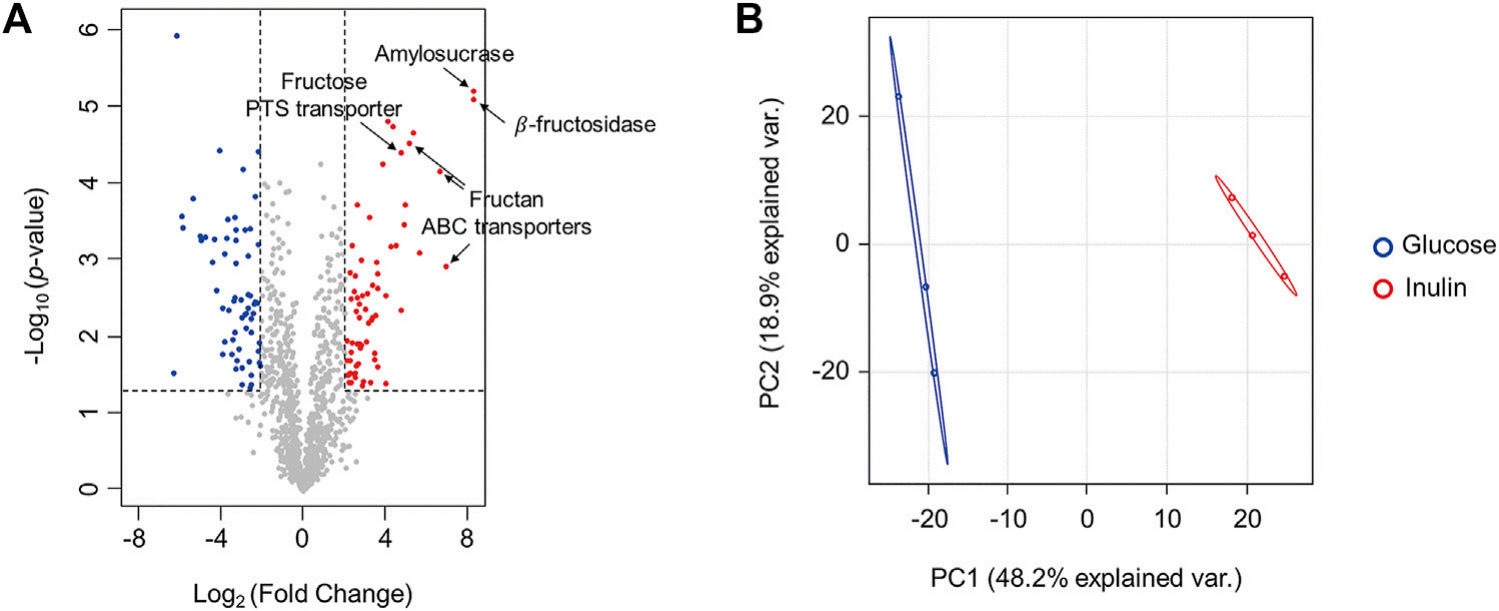
Comparison of proteomic changes of *F. prausnitzii* grown in mYCFA supplemented with glucose or inulin. **(A)** Volcano plot for proteomic changes of *F. prausnitzii* grown in mYCFA supplemented with glucose or inulin. Volcano plots indicate the upregulated proteins of inulin-grown *F. prausnitzii* in red circles and downregulated proteins in blue circles. Indications are proteins discussed in this study. **(B)** Principal component analysis (PCA) plot of proteomic analysis for *F. prausnitzii* grown in each carbon source. Proteomic analysis was performed in triplicates.

To investigate proteins involved in inulin utilization by *F. prausnitzii*, we investigated the intracellular proteomic changes in *F. prausnitzii* after culturing them using inulin or glucose, which showed growth potential as the sole carbon source. A total of 1,125 proteins were identified, of which 68 proteins were significantly upregulated (log_2_ (fold-change) >2, *p* < 0.05), and 62 proteins were significantly downregulated (log_2_ (fold-change) <-2, *p* < 0.05) during growth in inulin, compared to growth in glucose (Figure 3A, Supplementary Table S1). In addition, principal component analysis revealed that proteomic results of *F. prausnitzii* were significantly different when glucose and inulin were cultured as the sole carbon source (Figure 3B). Proteomic analysis showed that the function of upregulated proteins in inulin media was mainly associated with carbohydrate metabolism. In particular, glycoside hydrolases and ABC transporters were involved in inulin-type fructan degradation and intracellular transport, and the PTS transporter involved in fructose uptake were significantly upregulated.

### Glycoside Hydrolases Upregulated in *F. prausnitzii* Grown on Inulin

Proteomic analysis revealed that the most upregulated protein during growth in inulin compared to growth in glucose was a 261-fold upregulated enzyme, which belongs to glycoside hydrolase family 32 (GH32) (Figure 3A). A Basic Local Alignment Search Tool (BLAST) search for the amino acid sequence of this protein revealed ≥85% sequence identity with β-fructosidases (levanase/invertase), sucrose-6-phosphate hydrolase (sacA), and β-fructofuranosidase of *F. prausnitzii* L2-6, *F. prausnitzii,* and *Gemmiger formicilis*, respectively (Supplementary Table S2). All of them are hydrolytic enzymes belonging to the GH32 family and function as β-fructosidase (EC 3.2.1.26), which hydrolyzes the β-D-fructofuranoside bond of sucrose or inulin-type fructan and degrades them into fructose monosaccharides. A BLAST search also confirmed that the query sequence of this protein was highly conserved with the aligned sequences even in other *F. prausnitzii* strains (Supplementary Table S3). In addition, the second upregulated protein was an α-amylase catalytic domain protein that was upregulated 260-fold during growth in inulin compared to growth in glucose. A BLAST search revealed a sequence identity of ≥98% with amylosucrase (ams) and the degree of conservation of this protein among different *F. prausnitzii* strains (Supplementary Tables S4, S5). Amylosucrase (EC 2.4.1.4) is a hydrolase belonging to the GH13 family and is a glucose transference, which cleaves sucrose into glucose and fructose and transfers glucosyl residues to the glucan chain to form α-1,4-linked glucan (Montalk et al., 1999; Tian et al., 2018). Therefore, putative β-fructosidase and amylosucrase are enzymes capable of hydrolyzing inulin-type fructan and sucrose. However, while amylosucrase is a hydrolase, which uses sucrose as a substrate, β-fructosidase could degrade both the β-2,1 fructose-glucose bond and the β-2,1 bond between fructose, when a polymer such as inulin-type fructan is present as the substrate. β-fructosidase (EC 3.2.1.26) of *Thermotoga maritima* can release fructose from the inulin terminus by an exo-type mechanism and form sucrose (Liebl et al., 1998). In contrast, β-fructofuranosidase (EC 3.2.1.26) of *Bifidobacterium breve* UCC2003 cleaves the β-2,1 bond of glucose-fructose in sucrose and fructooligosaccharide (FOS) substrates, but not the β-2,1 bond between fructose (Ryan et al., 2005). Thus, even the same enzyme may have different functions depending on the source species and the substrate on which it acts. Therefore, we attempted to characterize the exact function of putative β-fructosidase and amylosucrase of *F. prausnitzii* by analyzing the enzyme activity and substrate specificity.

### Intracellular Inulin-type Fructan Degrading Enzymes Upregulated in *F. prausnitzii* Grown on Inulin

**FIGURE 4:**
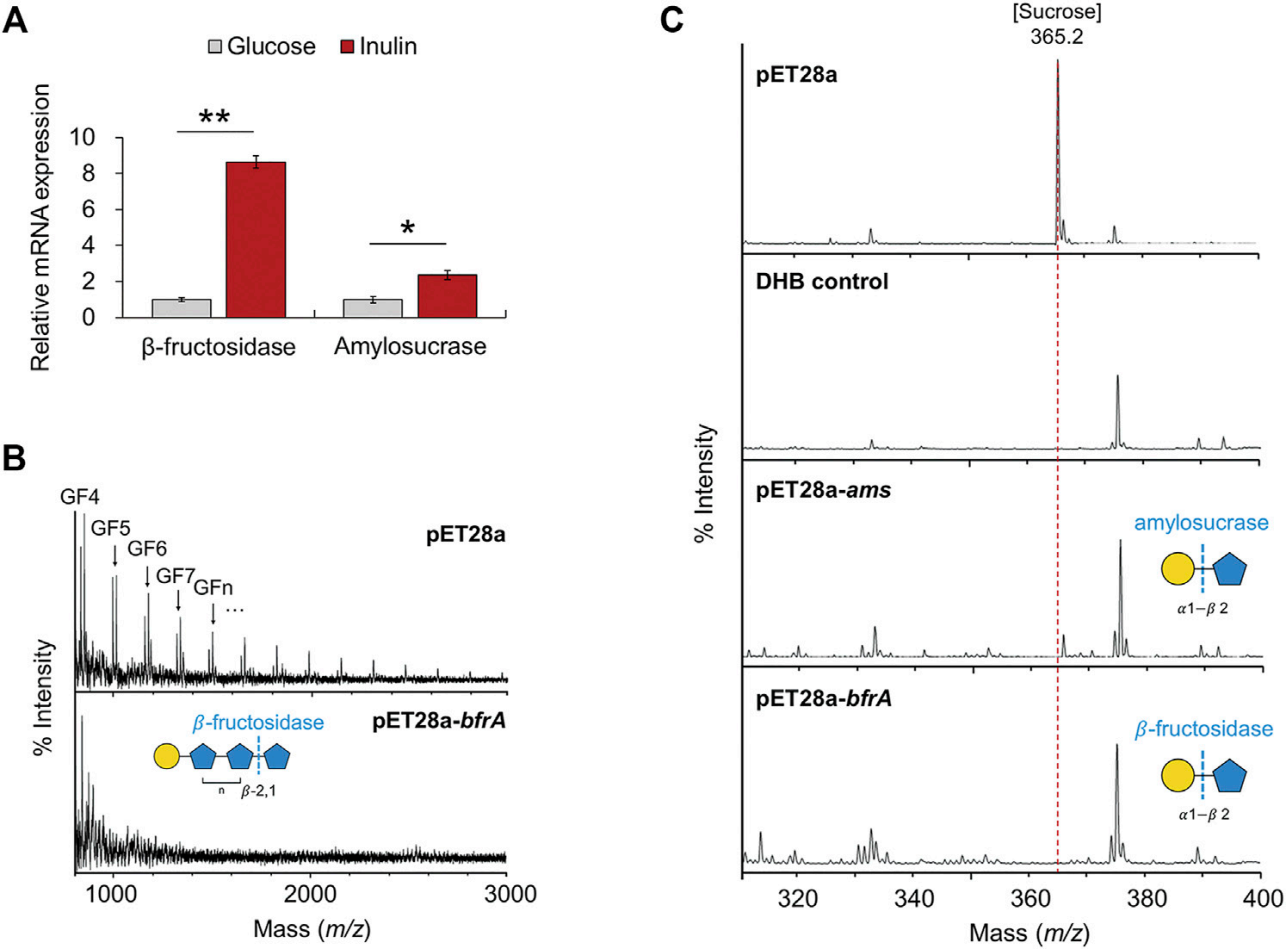
Mass spectrometry analysis of cell lysates. **(A)** mRNA expression of glycoside hydrolases (β-fructosidase, amylosucrase). The error bars indicate the standard deviation of triplicate samples. *p < 0.05, **p < 0.01. **(B)** MALDI-TOF-MS spectra of inulin degraded by cell lysates of pET28a-*bfrA*. Peaks are labeled by DP. G, glucose; F, fructose. All molecular ions are [M + Na]^+^. **(C)** MALDI-TOF-MS spectra of sucrose degraded by cell lysates of pET28a-*ams*, and pET28a-*bfrA*. All molecular ions are [M + Na]^+^.

To characterize putative β-fructosidase and amylosucrase upregulated during the growth of *F. prausnitzii* on inulin, we investigated mRNA expression levels of the genes encoding each hydrolase, using quantitative real time polymerase chain reaction analysis. The mRNA expression levels of β-fructosidase and amylosucrase significantly increased by 8.64-fold and 2.35-fold, respectively, during growth in inulin, compared to the levels observed during growth in glucose, similar to the data obtained from proteome analysis (Figure 4A). In addition, to evaluate the degradation activity of these enzymes on inulin and sucrose substrates, we cloned *bfrA* and *ams* into the pET28a vector and transformed them into *E. coli* BL21. The activity of the purified enzyme obtained after overexpression in *E. coli* was determined by treating the extracted cell lysate with inulin and sucrose, followed by analysis of the final product using matrix-assisted laser desorption/ionization-time of flight mass spectrometry. It was found that all lengths of inulin (DP = 2–60) were hydrolyzed by the pET28a-*bfrA* recombinant strain lysate regardless of the DP (Figure 4B). Thus, the putative β-fructosidase could cleave all of the β-2,1 bonds between the fructose of inulin-type fructan. In addition, sucrose was degraded by the pET28a-*ams* and pET28a-*bfrA* recombinant strains (Figure 4C). These results showed that putative amylosucrase and putative β-fructosidase were enzymes capable of cleaving the α1- β2 bond between α-D-glucose and β-D-fructose. However, although we established the hydrolytic activity of the two hydrolases on inulin-type fructan and sucrose through the recombinant strains, it remains unclear whether the degradation in the metabolic pathway of the enzyme was intracellular or extracellular.

**FIGURE 5:**
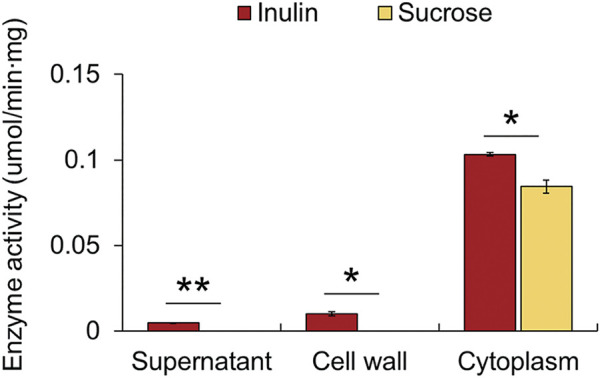
Fructan hydrolase enzyme activities in three fractions of the cell: cell-free supernatant, cell wall extract, and cytoplasmic extract. The activity was measured by determining the amount of fructose released per min per mg of protein. The error bars indicate the standard deviation of duplicate samples. *p < 0.05, **p < 0.01.

It is predicted that many cell wall-associated proteins of Gram-positive bacteria are anchored to the cell wall through the LPxTG (where x is any amino acid) cell wall anchor motif located in the C-terminal region of the amino acid sequence (Navarre and Schneewind, 1994). A cell wall anchor motif is present in the β-fructosidase of *Lactiplantibacillus plantarum* P14 and P76 and *Lacticaseibacillus paracasei* 1,195, and it has been confirmed that a cell wall-anchored β-fructosidase degrades fructan outside the cell (Goh et al., 2007; Buntin et al., 2017). In contrast, it is known that β-fructosidase of *Lactobacillus acidophilus* NCFM is a cytoplasmic protein, since it does not contain the LPxTG sequence and hence is expected to be an intracellular enzyme (Barrangou et al., 2003). However, we could not identify a specific sequence corresponding to the characteristic cell wall anchor motif from the amino acid sequences of β-fructosidase and amylosucrase (data not shown). Therefore, it could be predicted that both these enzymes are intracellular, which degrade inulin-type fructan transported into cells. Additionally, subcellular localization of the protein through PSORT Version 3.0.3 revealed that the two hydrolases were >99.7% cytoplasmic (Yu et al., 2010). To determine the location of the hydrolases more accurately, *F. prausnitzii*, cultured in inulin as a sole carbon source, was extracted by dividing it into three fractions: cell-free supernatant, cell wall extract, and cytoplasmic extract, and the amount of fructose released was measured when inulin and sucrose were used as substrates. When inulin was used as a substrate, the cytoplasmic extract showed 22 times more hydrolytic activity than the supernatant and 10 times more hydrolytic activity than the cell wall extract; when sucrose was used as a substrate, only the cytoplasmic extract showed activity (Figure 5). Therefore, it was confirmed that the enzyme that degraded inulin-type fructan in inulin metabolism of *F. prausnitzii* was an intracellular enzyme, which degrades inulin-type fructan transported into the cell.

### Uptake of Inulin-Type Fructans by Upregulated Fructan ABC Transporters in *F. prausnitzii* Grown on Inulin

**FIGURE 6:**
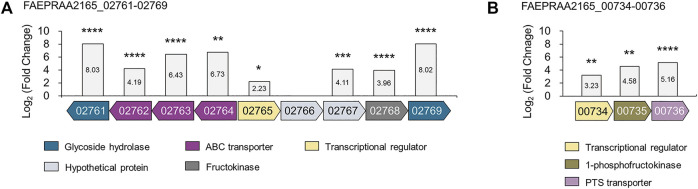
The gene organization of proteins upregulated from proteomic analyses of *F. prausnitzii* grown on inulin relative to glucose. Proteins involved in **(A)** inulin-type fructan utilization and **(B)** fructose uptake were annotated. The locus tag numbers FAEPRAA2165_XXXXX are abbreviated with the last numbers after the hyphen. Proteins with similar predicted functions are colored by the same color. The log_2_ (fold-change) relative to *F. prausnitzii* grown on glucose is shown on the *y*-axis. The proteomic analysis was performed in triplicates. *p < 0.05, **p < 0.01, ***p < 0.001, ****p < 0.0001., Protein annotation: β-fructosidase (02761); fructan ABC transporters (02762–02764); LacI family transcriptional regulator (02765); hypothetical proteins (02766–02767); fructokinase (02768); amylosucrase (02769); DeoR family transcriptional regulator (00734); 1-phosphofructokinase (00735); fructose PTS transporter (00736).

From the proteomic results, we confirmed that glycoside hydrolases and proteins involved in the intracellular transport of sugars were upregulated 18-fold or more during growth in inulin (Figures 6A,B). A BLAST search confirmed that these proteins were ABC transporters, which transport substrates using ATP as an energy source, and the PTS transporter that transports sugar into cells and simultaneously phosphorylates it (Supplementary Table S7) (Saurin et al., 1999; Deutscher et al., 2006). The metabolism of sugar using transport systems, such as ABC and PTS transporters, generally follows two metabolic pathways. Interestingly, various studies have reported that transport systems are involved in inulin-type fructan utilization together with β-fructosidase in various probiotic bacteria. First, inulin-type fructan enters the cell, in an undigested state through the transporter, and is degraded by the cytoplasmic enzyme, which can be mainly taken up by the ABC transporter in this case. Tsujikawa et al. reported that *Lactobacillus delbrueckii* JCM 1002T transported inulin (DP > 8) into cells in an undigested state through the inulin ABC transporter (Tsujikawa et al., 2013; Tsujikawa et al., 2021). In addition, the ABC transport system of *Lactobacillus acidophilus* NCFM is involved in FOS (DP 2–4) utilization, together with intracellular β-fructosidase (*bfrA*) (Barrangou et al., 2003). Second, after the substrate is degraded by enzymes present in the extracellular space or the cell wall, the product enters the cell through the PTS transporter. Goh Yong et al. showed that when *Lactobacillus paracasei* 1,195 degrades FOS (DP < 10) extracellularly through cell wall-anchored β-fructosidase, the generated fructose and sucrose are taken up into cells through each PTS transporter (Goh et al., 2006; Goh et al., 2007). In addition, in fructan utilization by *Roseburia inulinivorans* A2-194, inulin-type fructan (DP 2–60) is transported into cells by the ABC transporter and degraded by β-fructofuranosidase, while the PTS transporter is reported to be involved in the uptake of fructose (Scott et al., 2011). This study confirmed the intracellular activity of β-fructosidase of *F. prausnitzii* using an enzyme assay. Therefore, it is predicted that the inulin-type fructan enters into cells in its original state through the putative ABC transporter and is then degraded by cytoplasmic β-fructosidase.

Furthermore, bacterial carbohydrate utilization is generally caused by the co-regulation of genes encoding carbohydrate hydrolases, transporters, and transcriptional regulators in adjacent positions (Barrangou et al., 2003; Goh et al., 2007). We found that the location of the genes encoding β-fructosidase, amylosucrase, and putative ABC transporters were located adjacent to those of the putative fructokinase and putative LacI family regulator (locus tag FAEPRAA2165_02761-02769), which were upregulated 15.56-fold and 4.69-fold, respectively, during growth in inulin (Figure 6A, Supplementary Table S7). Interestingly, these proteins had a similar gene organization to β-fructofuranosidase, ABC transporters, 6-phosphofructokinase, and LacI family regulators, belonging to the fructan utilization gene cluster induced during inulin growth in *R. inulinivorans*. Especially, β-fructofuranosidase, and three ABC transport system components shared 45–66% identity with β-fructofuranosidase and ABC transporters in this study (Supplementary Table S8). Accordingly, we suggest that *F. prausnitzii* utilizes inulin-type fructan (DP 2–60) by uptake into cells through fructan ABC transporter and degradation by cytoplasmic β-fructosidase, similar to fructan utilization by *R. inulinivorans*. However, further research is needed to determine the exact DP of inulin-type fructan taken up into the cell through the fructan ABC transporter.

### Upregulated Fructose Uptake Ability in *F. prausnitzii* Grown on Inulin

**FIGURE 7:**
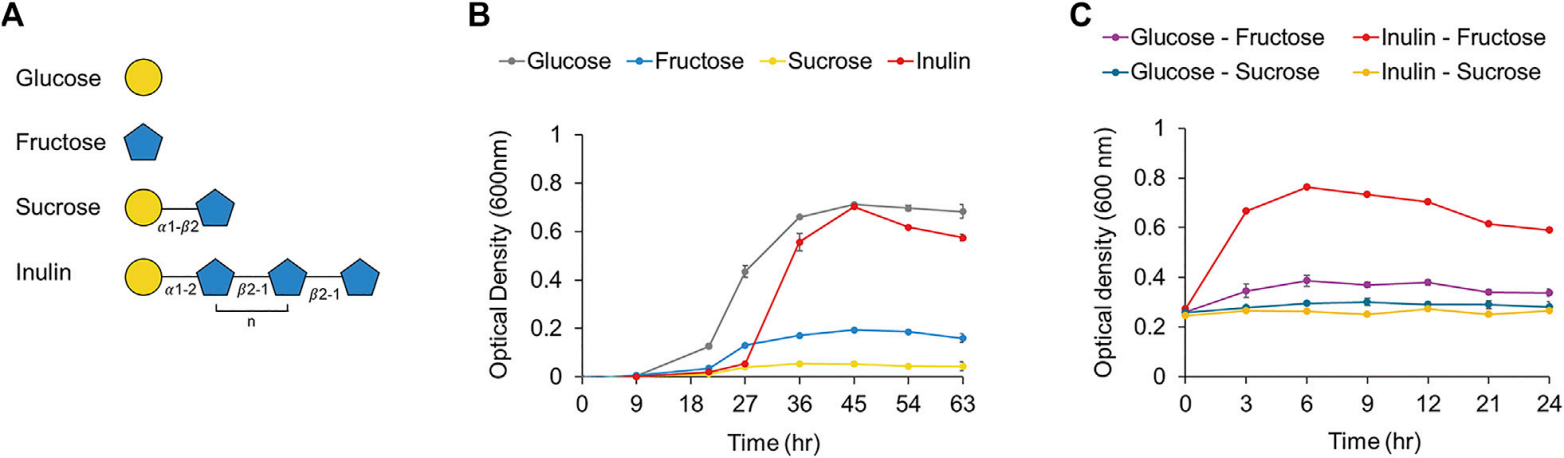
**(A)** Structures of the components (i.e., glucose, fructose, sucrose, inulin). of inulin were shown. **(B)** The growth curve of *F. prausnitzii* cultured in mYCFA supplemented with either glucose, fructose, sucrose, or inulin. **(C)** The growth curve of *F. prausnitzii* cultured in mYCFA supplemented with either fructose or sucrose. Cells cultured in inulin or glucose were harvested and inoculated in fructose or sucrose. Each point on the curves represents the average of three independent experiments. Error bars represent standard deviations (s.d.).

We confirmed that the putative PTS transporter was upregulated 35-fold in *F. prausnitzii* grown on inulin (Figure 6B). A BLAST search confirmed that this transporter responds specifically to fructose; a recent study has reported that a fructose-specific PTS transporter participates in the intracellular transport of fructose and is induced when *F. prausnitzii* is grown in the presence of fructose (Supplementary Table S7) (Kang et al., 2021). In addition, proteomic data confirmed that the transcriptional regulator and 1-phosphofructokinase encoded by the gene adjacent to the gene of the fructose PTS transporter were upregulated (Figure 6B). Because the PTS transporter transports sugar into the cell and simultaneously phosphorylates it, *F. prausnitzii* is expected to take up fructose into the cell through the fructose PTS transporter and phosphorylate it to fructose 1-phosphate, which is subsequently converted into fructose 1,6-bisphosphate by 1-phosphofructokinase and enters the glycolytic pathway (Kang et al., 2021). To explore the activity of the fructose PTS transporter, we compared the growth ability of *F. prausnitzii* on glucose, fructose, sucrose, and inulin produced during the digestion process of inulin (Figure 7A). As a result, *F. prausnitzii* showed a significant growth ability on glucose and inulin substrates, but a relatively low growth ability in fructose, and growth was difficult in sucrose (Figure 7B). These results demonstrated that *F. prausnitzii* preferred to utilize inulin, which has a higher DP than fructose and sucrose, and the transport of inulin-type fructan through the fructan ABC transporter was more dominant than that of fructose through the fructose PTS transporter. In addition, to investigate whether inulin induced upregulation of the fructose PTS transporter in *F. prausnitzii* results in increased uptake of fructose by cells, we inoculated *F. prausnitzii*, isolated in the mid-log phase grown on glucose or inulin, to a new medium supplemented with fructose and sucrose, and compared the growth. The growth ability of inulin-grown *F. prausnitzii* on fructose was significantly higher than that of glucose-grown *F. prausnitzii*. (Figure 7C). These results suggested that when *F. prausnitzii* utilized inulin, and the fructose PTS transporter was upregulated, thereby enhancing the ability to uptake fructose into cells. Interestingly, *F. prausnitzii* could not uptake sucrose despite the upregulation of the fructose PTS transporter and fructan ABC transporter. These results revealed that sucrose could not be transported through the fructose PTS transporter because it was not degraded into fructose outside the cell and was not directly transported into the cell even in the state of undigested sucrose. In summary, we demonstrated that when *F. prausnitzii* metabolized inulin, the fructose PTS transporter, which can specifically uptake fructose into the cell, was upregulated; however, we confirmed that the transport of inulin-type fructan by the ABC transporter was more dominant. This suggested that the main pathway for inulin utilization in *F. prausnitzii* was the selective transport of inulin-type fructan into the cell through the fructan ABC transporter and the degradation of inulin-type fructan into fructose by the cytoplasmic degrading enzyme.

### Metabolomic Changes of *F. prausnitzii* Grown on Inulin

We compared quantitative changes in the intracellular metabolome of *F. prausnitzii* incubated with inulin or glucose as the sole carbon source, to observe changes in metabolites related to inulin utilization in *F. prausnitzii*. In total, 129 metabolite changes were observed, and when inulin was grown as a sole carbon source, 21 metabolites were significantly upregulated (fold-change >1.5, *p* < 0.05), and 51 metabolites were downregulated (fold-change < −1.5, *p* < 0.05) (Supplementary Figure S2, Supplementary Table S9). Proteomic data have earlier revealed that when *F. prausnitzii* grows on inulin, the fructose utilization system is also activated. Thus, it could be inferred that fructokinase phosphorylates fructose, which is produced by the degradation of inulin-type fructan that is transported inside the cell by fructan ABC transporter, and converts it into fructose-6-phosphate. In addition, 1-phosphofructokinase converts phosphorylated fructose, which is produced by the fructose PTS transporter while taken up into fructose 1,6-bisphosphate, which could be metabolized to the glycolytic pathway. Metabolomic analysis showed that fructose-6-phosphate was significantly upregulated 1.89-fold when *F. prausnitzii* was grown on inulin compared to that obtained with growth on glucose (Table 1). In addition, glucose-6-phosphate, which is produced by the phosphorylation of intracellular glucose and is converted into fructose-6-phosphate to enter the glycolysis pathway, was also significantly upregulated by 1.85 times. This indicated that the increase in intracellular fructose through inulin-type fructan metabolism was eventually induced in the glycolysis pathway and the tricarboxylic acid (TCA) cycle for energy production, which is essential for cell growth. In addition, the metabolite expression pattern of the TCA cycle, which is important for the production of ATP and the chemical energy produced by carbohydrate metabolism, was quantitatively confirmed. It was found that citrate, aconitate, and succinyl-CoA were upregulated by 2.35, 1.98, and 1.89-fold, respectively; malate, succinate, and acetyl-CoA were downregulated by 0.26, 0.30, and 0.43-fold, respectively. In addition, ATP decreased by 0.63-fold, which is believed to result in relative differences in ATP due to the energy consumption required for inulin metabolism by *F. prausnitzii* compared to the growth of *F. prausnitzii* on glucose. However, these results suggest that inulin can be selectively used as a carbohydrate source by *F. prausnitzii* in a targeted manner compared to glucose, which other gut bacterial species can utilize.

**TABLE 1:** The metabolomic changes involved in the energy metabolism of *F. prausnitzii* according to fermentation of inulin or glucose. Data are presented with different four samples (n = 4) by LC-MS/MS. *p < 0.05, **p < 0.01, ***p < 0.001 by two-tailed Student’s t-test.

Name	Log_2_FC (Inulin/Glucose)
Citrate	1.23^***^
Aconitate	0.98^*^
Fructose 6-phosphate	0.92^*^
Succinyl-CoA	0.92^*^
Glucose 6-phosphate	0.89^*^
Fructose 1,6-bisphosphate	−0.6^**^
Adenosine triphosphate	−0.66^***^
Acetyl-CoA	−1.22^**^
Succinate	−1.76^***^
Malate	−1.92^***^

## Conclusion

**FIGURE 8:**
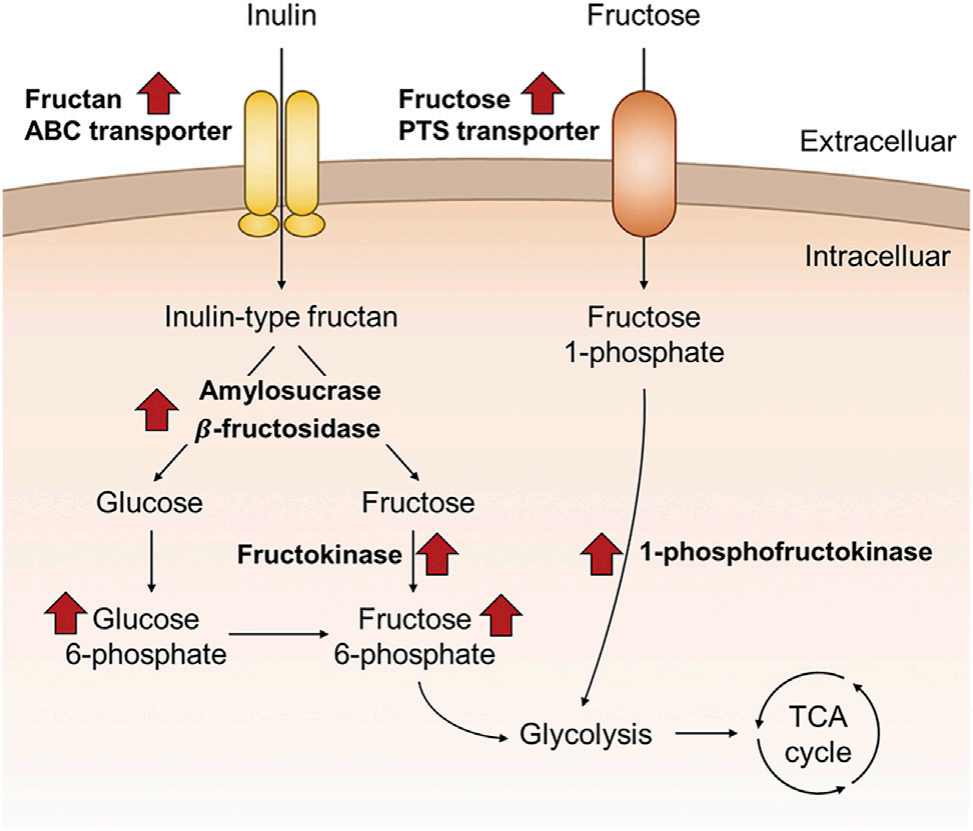
Schematic overview of the proposed inulin utilization pathway of *F. prausnitzii* that were discovered in this study, based on the proteomic and metabolomic data of *F. prausnitzii* grown on inulin relative to glucose.

Based on a molecular perspective, this study attempted to elucidate the biological mechanism whereby *F. prausnitzii*, a human health-promoting intestinal butyrate-producing bacterium, can selectively digest prebiotic inulin. *F. prausnitzii* could selectively digest inulin among various dietary fibers, and when grown in inulin as the sole carbon source, it has an excellent butyrate production capacity of about 65% of that when grown on glucose. In addition, intracellular proteomic data revealed that proteins involved in inulin-type fructan degradation and intracellular transport were upregulated in *F. prausnitzii* grown on inulin. First, β-fructosidase and amylosucrase, which can completely degrade inulin-type fructan, were significantly upregulated, and the activity of enzymes against fructan and sucrose degradation was verified in *E. coli* using genetic recombination. In addition, analysis of the subcellular location of enzymes demonstrated that the inulin-type fructan degrading enzymes were present intracellularly. We found that fructan ABC transporters, which could directly uptake inulin-type fructan into cells for intracellular degradation, were upregulated. In addition, the fructose transporter transporting fructose into cells was upregulated, implying that *F. prausnitzii* improved not only inulin-type fructan utilization but also the fructose uptake ability through inulin metabolism. In addition, intracellular metabolomics confirmed that *F. prausnitzii* efficiently used fructose, the final degradation product of inulin-type fructan, for energy production during growth using inulin as a carbon source. In conclusion, we conducted a study by a multiomics approach to identify the proteins involved in inulin utilization by *F. prausnitzii* and were able to present a molecular scale model of *F. prausnitzii* utilizing inulin in the intestine (Figure 8). Thus, the results have potential applications in the selection and development of optimized prebiotics in the future to enhance human health by improving the survival of *F. prausnitzii*, which has useful metabolic productivity as compared to other intestinal microorganisms.

## Data Availability

The mass spectrometry proteomics data generated in this study have been deposited in the ProteomeXchange Consortium via the PRIDE (Perez-Riverol et al., 2019) partner repository with the dataset identifier “PXD030078”.

## References

[B1] Bäckhed F. Ley R. E. Sonnenburg J. L. Peterson D. A. Gordon J. I. (2005). Host-bacterial Mutualism in the Human Intestine. Science 307 (5717), 1915–1920. 10.1126/science.1104816 15790844

[B2] Barrangou R. Altermann E. Hutkins R. Cano R. Klaenhammer T. R. (2003). Functional and Comparative Genomic Analyses of an Operon Involved in Fructooligosaccharide Utilization by Lactobacillus Acidophilus. Proc. Natl. Acad. Sci. 100 (15), 8957–8962. 10.1073/pnas.1332765100 12847288 PMC166420

[B3] Buntin N. Hongpattarakere T. Ritari J. Douillard F. P. Paulin L. Boeren S. (2017). An Inducible Operon Is Involved in Inulin Utilization in Lactobacillus Plantarum Strains, as Revealed by Comparative Proteogenomics and Metabolic Profiling. Appl. Environ. Microbiol. 83 (2), e02402-16. 10.1128/AEM.02402-16 27815279 PMC5203619

[B4] Campbell J. M. Fahey G. C. Jr. Wolf B. W. (1997). Selected Indigestible Oligosaccharides Affect Large Bowel Mass, Cecal and Fecal Short-Chain Fatty Acids, pH and Microflora in Rats. J. Nutr. 127 (1), 130–136. 10.1093/jn/127.1.130 9040556

[B5] Cox J. Hein M. Y. Luber C. A. Paron I. Nagaraj N. Mann M. (2014). Accurate Proteome-wide Label-free Quantification by Delayed Normalization and Maximal Peptide Ratio Extraction, Termed MaxLFQ. Mol. Cell Proteomics 13 (9), 2513–2526. 10.1074/mcp.M113.031591 24942700 PMC4159666

[B6] Desai M. S. Seekatz A. M. Koropatkin N. M. Kamada N. Hickey C. A. Wolter M. (2016). A Dietary Fiber-Deprived Gut Microbiota Degrades the Colonic Mucus Barrier and Enhances Pathogen Susceptibility. Cell 167 (5), 1339–1353. e1321. 10.1016/j.cell.2016.10.043 27863247 PMC5131798

[B7] Deutscher J. Francke C. Postma P. W. (2006). How Phosphotransferase System-Related Protein Phosphorylation Regulates Carbohydrate Metabolism in Bacteria. Microbiol. Mol. Biol. Rev. 70 (4), 939–1031. 10.1128/mmbr.00024-06 17158705 PMC1698508

[B8] Dewulf E. M. Cani P. D. Claus S. P. Fuentes S. Puylaert P. G. Neyrinck A. M. (2013). Insight into the Prebiotic Concept: Lessons from an Exploratory, Double Blind Intervention Study with Inulin-type Fructans in Obese Women. Gut 62 (8), 1112–1121. 10.1136/gutjnl-2012-303304 23135760 PMC3711491

[B9] Duncan S. H. Hold G. L. Harmsen H. J. M. Stewart C. S. Flint H. J. (2002). Growth Requirements and Fermentation Products of Fusobacterium Prausnitzii, and a Proposal to Reclassify it as Faecalibacterium Prausnitzii Gen. nov., Comb. Nov. Int. J. Syst. Evol. Microbiol. 52 (6), 2141–2146. 10.1099/00207713-52-6-2141 12508881

[B10] Eckburg P. B. Bik E. M. Bernstein C. N. Purdom E. Dethlefsen L. Sargent M. (2005). Diversity of the Human Intestinal Microbial flora. Science 308 (5728), 1635–1638. 10.1126/science.1110591 15831718 PMC1395357

[B11] Fang S. Yan B. Tian F. Lian H. Zhao J. Zhang H. (2021). β-Fructosidase FosE Activity in Lactobacillus Paracasei Regulates Fructan Degradation during Sourdough Fermentation and Total FODMAP Levels in Steamed Bread. LWT 145, 111294. 10.1016/j.lwt.2021.111294

[B12] Fitzgerald C. B. Shkoporov A. N. Sutton T. D. S. Chaplin A. V. Velayudhan V. Ross R. P. (2018). Comparative Analysis of Faecalibacterium Prausnitzii Genomes Shows a High Level of Genome Plasticity and Warrants Separation into New Species-Level Taxa. BMC Genomics 19 (1), 931. 10.1186/s12864-018-5313-6 30547746 PMC6295017

[B13] Flint H. J. Scott K. P. Louis P. Duncan S. H. (2012). The Role of the Gut Microbiota in Nutrition and Health. Nat. Rev. Gastroenterol. Hepatol. 9 (10), 577–589. 10.1038/nrgastro.2012.156 22945443

[B14] Fung K. Y. C. Cosgrove L. Lockett T. Head R. Topping D. L. (2012). A Review of the Potential Mechanisms for the Lowering of Colorectal Oncogenesis by Butyrate. Br. J. Nutr. 108 (5), 820–831. 10.1017/s0007114512001948 22676885

[B15] Gibson G. R. Roberfroid M. B. (1995). Dietary Modulation of the Human Colonic Microbiota: Introducing the Concept of Prebiotics. J. Nutr. 125 (6), 1401–1412. 10.1093/jn/125.6.1401 7782892

[B16] Goh Y. J. Lee J.-H. Hutkins R. W. (2007). Functional Analysis of the Fructooligosaccharide Utilization Operon in Lactobacillus Paracasei 1195. Appl. Environ. Microbiol. 73 (18), 5716–5724. 10.1128/AEM.00805-07 17644636 PMC2074902

[B17] Goh Y. J. Zhang C. Benson A. K. Schlegel V. Lee J.-H. Hutkins R. W. (2006). Identification of a Putative Operon Involved in Fructooligosaccharide Utilization by Lactobacillus Paracasei. Appl. Environ. Microbiol. 72 (12), 7518–7530. 10.1128/AEM.00877-06 17028235 PMC1694223

[B18] Hamer H. M. Jonkers D. Venema K. Vanhoutvin S. Troost F. J. Brummer R. J. (2008). Review Article: the Role of Butyrate on Colonic Function. Aliment. Pharmacol. Ther. 27 (2), 104–119. 10.1111/j.1365-2036.2007.03562.x 17973645

[B19] Holscher H. D. Doligale J. L. Bauer L. L. Gourineni V. Pelkman C. L. Fahey G. C. (2014). Gastrointestinal Tolerance and Utilization of Agave Inulin by Healthy Adults. Food Funct. 5 (6), 1142–1149. 10.1039/C3FO60666J 24664349

[B20] Jia W. Whitehead R. N. Griffiths L. Dawson C. Waring R. H. Ramsden D. B. (2010). Is the Abundance of Faecalibacterium Prausnitzii Relevant to Crohn's Disease. FEMS Microbiol. Lett. 310 (2), 138–144. 10.1111/j.1574-6968.2010.02057.x 20695899 PMC2962807

[B21] Jo S.-H. Song W.-S. Park H.-G. Lee J.-S. Jeon H.-J. Lee Y.-H. (2020). Multi-omics Based Characterization of Antibiotic Response in Clinical Isogenic Isolates of Methicillin-Susceptible/-Resistant *Staphylococcus aureus* . RSC Adv. 10 (46), 27864–27873. 10.1039/D0RA05407K PMC9055585 35516943

[B22] Joossens M. Huys G. Cnockaert M. De Preter V. Verbeke K. Rutgeerts P. (2011). Dysbiosis of the Faecal Microbiota in Patients with Crohn's Disease and Their Unaffected Relatives. Gut 60 (5), 631–637. 10.1136/gut.2010.223263 21209126

[B23] Kang D. Ham H. I. Lee S. H. Cho Y. J. Kim Y. R. Yoon C. K. (2021). Functional Dissection of the Phosphotransferase System Provides Insight into the Prevalence of Faecalibacterium Prausnitzii in the Host Intestinal Environment. Environ. Microbiol. 23 (8), 4726–4740. 10.1111/1462-2920.15681 34296500

[B24] Kaoutari A. E. Armougom F. Gordon J. I. Raoult D. Henrissat B. (2013). The Abundance and Variety of Carbohydrate-Active Enzymes in the Human Gut Microbiota. Nat. Rev. Microbiol. 11 (7), 497–504. 10.1038/nrmicro3050 23748339

[B25] Khan M. T. van Dijl J. M. Harmsen H. J. M. (2014). Antioxidants Keep the Potentially Probiotic but Highly Oxygen-Sensitive Human Gut Bacterium Faecalibacterium Prausnitzii Alive at Ambient Air. PLoS One 9 (5), e96097. 10.1371/journal.pone.0096097 24798051 PMC4010535

[B26] Koleva P. T. Valcheva R. S. Sun X. Gänzle M. G. Dieleman L. A. (2012). Inulin and Fructo-Oligosaccharides Have Divergent Effects on Colitis and Commensal Microbiota in HLA-B27 Transgenic Rats. Br. J. Nutr. 108 (9), 1633–1643. 10.1017/S0007114511007203 22243836

[B27] Liebl W. Brem D. Gotschlich A. (1998). Analysis of the Gene for β-fructosidase (Invertase, Inulinase) of the Hyperthermophilic Bacterium Thermotoga Maritima , and Characterisation of the Enzyme Expressed in *Escherichia coli* . Appl. Microbiol. Biotechnol. 50 (1), 55–64. 10.1007/s002530051256 9720201

[B28] Lopez-Siles M. Martinez-Medina M. Surís-Valls R. Aldeguer X. Sabat-Mir M. Duncan S. H. (2016). Changes in the Abundance of Faecalibacterium Prausnitzii Phylogroups I and II in the Intestinal Mucosa of Inflammatory Bowel Disease and Patients with Colorectal Cancer. Inflamm. Bowel Dis. 22 (1), 28–41. 10.1097/mib.0000000000000590 26595550

[B29] Louis P. Flint H. J. (2017). Formation of Propionate and Butyrate by the Human Colonic Microbiota. Environ. Microbiol. 19 (1), 29–41. 10.1111/1462-2920.13589 27928878

[B30] Martín R. Miquel S. Chain F. Natividad J. M. Jury J. Lu J. (2015). Faecalibacterium Prausnitzii Prevents Physiological Damages in a Chronic Low-Grade Inflammation Murine Model. BMC Microbiol. 15 (1), 67–12. 10.1186/s12866-015-0400-1 25888448 PMC4391109

[B31] Miquel S. Leclerc M. Martin R. Chain F. Lenoir M. Raguideau S. (2015). Identification of Metabolic Signatures Linked to Anti-inflammatory Effects of Faecalibacterium Prausnitzii. Mbio 6(2)**,** e00300-00315. 10.1128/mBio.00300-15 25900655 PMC4453580

[B32] Miquel S. Martín R. Bridonneau C. Robert V. Sokol H. Bermúdez-Humarán L. G. (2014). Ecology and Metabolism of the Beneficial Intestinal Commensal bacteriumFaecalibacterium Prausnitzii. Gut Microbes 5 (2), 146–151. 10.4161/gmic.27651 24637606 PMC4063839

[B33] Miquel S. Martín R. Rossi O. Bermúdez-Humarán L. Chatel J. Sokol H. (2013). Faecalibacterium Prausnitzii and Human Intestinal Health. Curr. Opin. Microbiol. 16 (3), 255–261. 10.1016/j.mib.2013.06.003 23831042

[B34] Moens F. De Vuyst L. (2017). Inulin-type Fructan Degradation Capacity ofClostridiumcluster IV and XIVa Butyrate-Producing colon Bacteria and Their Associated Metabolic Outcomes. Beneficial Microbes 8 (3), 473–490. 10.3920/bm2016.0142 28548573

[B35] Navarre W. W. Schneewind O. (1994). Proteolytic Cleavage and Cell wall Anchoring at the LPXTG Motif of Surface Proteins in Gram-Positive Bacteria. Mol. Microbiol. 14 (1), 115–121. 10.1111/j.1365-2958.1994.tb01271.x 7830549

[B36] Nyangale E. P. Mottram D. S. Gibson G. R. (2012). Gut Microbial Activity, Implications for Health and Disease: the Potential Role of Metabolite Analysis. J. Proteome Res. 11 (12), 5573–5585. 10.1021/pr300637d 23116228

[B37] Oren A. Garrity G. M. (2021). Valid Publication of the Names of Forty-Two Phyla of Prokaryotes. Int. J. Syst. Evol. Microbiol. 71 (10). 10.1099/ijsem.0.005056 34694987

[B38] O’Toole P. W. Marchesi J. R. Hill C. (2017). Next-generation Probiotics: the Spectrum from Probiotics to Live Biotherapeutics. Nat. Microbiol. 2 (5), 17057. 10.1038/nmicrobiol.2017.57 28440276

[B39] Pang Z. Chong J. Zhou G. de Lima Morais D. A. Chang L. Barrette M. (2021). MetaboAnalyst 5.0: Narrowing the gap between Raw Spectra and Functional Insights. Nucleic Acids Res. 49 (W1), W388–W396. 10.1093/nar/gkab382 34019663 PMC8265181

[B40] Perez-Riverol Y. Csordas A. Bai J. Bernal-Llinares M. Hewapathirana S. Kundu D. J. (2019). The PRIDE Database and Related Tools and Resources in 2019: Improving Support for Quantification Data. Nucleic Acids Res. 47 (D1), D442–D450. 10.1093/nar/gky1106 30395289 PMC6323896

[B41] Potocki De Montalk G. Remaud-Simeon M. Willemot R. M. Planchot V. Monsan P. (1999). Sequence Analysis of the Gene Encoding Amylosucrase from Neisseria Polysaccharea and Characterization of the Recombinant Enzyme. J. Bacteriol. 181 (2), 375–381. 10.1128/jb.181.2.375-381.1999 9882648 PMC93388

[B42] Ramirez-Farias C. Slezak K. Fuller Z. Duncan A. Holtrop G. Louis P. (2008). Effect of Inulin on the Human Gut Microbiota: Stimulation ofBifidobacterium adolescentisandFaecalibacterium Prausnitzii. Br. J. Nutr. 101 (4), 541–550. 10.1017/S0007114508019880 18590586

[B43] Roberfroid M. B. Delzenne N. M. (1998). Dietary Fructans. Annu. Rev. Nutr. 18, 117–143. 10.1146/annurev.nutr.18.1.117 9706221

[B44] Roberfroid M. B. (2005). Introducing Inulin-type Fructans. Br. J. Nutr. 93 (Suppl. 1), S13–S25. 10.1079/bjn20041350 15877886

[B45] Roediger W. E. (1980). Role of Anaerobic Bacteria in the Metabolic Welfare of the Colonic Mucosa in Man. Gut 21 (9), 793–798. 10.1136/gut.21.9.793 7429343 PMC1419533

[B46] Ryan S. M. Fitzgerald G. F. van Sinderen D. (2005). Transcriptional Regulation and Characterization of a Novel β-Fructofuranosidase-Encoding Gene from Bifidobacterium Breve UCC2003. Appl. Environ. Microbiol. 71 (7), 3475–3482. 10.1128/AEM.71.7.3475-3482.2005 16000751 PMC1169055

[B47] Saurin W. Hofnung M. Dassa E. (1999). Getting in or Out: Early Segregation between Importers and Exporters in the Evolution of ATP-Binding Cassette (ABC) Transporters. J. Mol. Evol. 48 (1), 22–41. 10.1007/pl00006442 9873074

[B48] Scott K. P. Martin J. C. Chassard C. Clerget M. Potrykus J. Campbell G. (2011). Substrate-driven Gene Expression in Roseburia Inulinivorans: Importance of Inducible Enzymes in the Utilization of Inulin and Starch. Proc. Natl. Acad. Sci. 108 (Suppl. 1), 4672–4679. 10.1073/pnas.1000091107 20679207 PMC3063597

[B49] Shah A. D. Goode R. J. A. Huang C. Powell D. R. Schittenhelm R. B. (2020). LFQ-analyst: An Easy-To-Use Interactive Web Platform to Analyze and Visualize Label-free Proteomics Data Preprocessed with MaxQuant. J. Proteome Res. 19 (1), 204–211. 10.1021/acs.jproteome.9b00496 31657565

[B50] Sokol H. Pigneur B. Watterlot L. Lakhdari O. Bermúdez-Humarán L. G. Gratadoux J.-J. (2008). Faecalibacterium Prausnitzii Is an Anti-inflammatory Commensal Bacterium Identified by Gut Microbiota Analysis of Crohn Disease Patients. Proc. Natl. Acad. Sci. 105 (43), 16731–16736. 10.1073/pnas.0804812105 18936492 PMC2575488

[B51] Song W.-S. Park H.-G. Kim S.-M. Jo S.-H. Kim B.-G. Theberge A. B. (2020). Chemical Derivatization-Based LC-MS/MS Method for Quantitation of Gut Microbial Short-Chain Fatty Acids. J. Ind. Eng. Chem. 83, 297–302. 10.1016/j.jiec.2019.12.001

[B52] Tian Y. Xu W. Zhang W. Zhang T. Guang C. Mu W. (2018). Amylosucrase as a Transglucosylation Tool: From Molecular Features to Bioengineering Applications. Biotechnol. Adv. 36 (5), 1540–1552. 10.1016/j.biotechadv.2018.06.010 29935268

[B53] Tsujikawa Y. Ishikawa S. Sakane I. Yoshida K.-I. Osawa R. (2021). Identification of Genes Encoding a Novel ABC Transporter in Lactobacillus Delbrueckii for Inulin Polymers Uptake. Sci. Rep. 11 (1), 16007. 10.1038/s41598-021-95356-1 34362962 PMC8346543

[B54] Tsujikawa Y. Nomoto R. Osawa R. (2013). Difference in Degradation Patterns on Inulin-type Fructans Among Strains of Lactobacillus Delbrueckii and Lactobacillus Paracasei. Biosci. Microbiota Food Health 32 (4), 157–165. 10.12938/bmfh.32.157 24936375 PMC4034334

[B55] Tyanova S. Temu T. Sinitcyn P. Carlson A. Hein M. Y. Geiger T. (2016). The Perseus Computational Platform for Comprehensive Analysis of (Prote)omics Data. Nat. Methods 13 (9), 731–740. 10.1038/nmeth.3901 27348712

[B56] Ueda A. Shinkai S. Shiroma H. Taniguchi Y. Tsuchida S. Kariya T. (2021). Identification of Faecalibacterium Prausnitzii Strains for Gut Microbiome-Based Intervention in Alzheimer's-type Dementia. Cel Rep. Med. 2 (9), 100398. 10.1016/j.xcrm.2021.100398 PMC8484692 34622235

[B57] Varela E. Manichanh C. Gallart M. Torrejón A. Borruel N. Casellas F. (2013). Colonisation byFaecalibacterium Prausnitziiand Maintenance of Clinical Remission in Patients with Ulcerative Colitis. Aliment. Pharmacol. Ther. 38 (2), 151–161. 10.1111/apt.12365 23725320

[B58] Walker A. W. Ince J. Duncan S. H. Webster L. M. Holtrop G. Ze X. (2011). Dominant and Diet-Responsive Groups of Bacteria within the Human Colonic Microbiota. ISME J. 5 (2), 220–230. 10.1038/ismej.2010.118 20686513 PMC3105703

[B59] Wang H.-B. Wang P.-Y. Wang X. Wan Y.-L. Liu Y.-C. (2012). Butyrate Enhances Intestinal Epithelial Barrier Function via Up-Regulation of Tight junction Protein Claudin-1 Transcription. Dig. Dis. Sci. 57 (12), 3126–3135. 10.1007/s10620-012-2259-4 22684624

[B60] Willing B. P. Dicksved J. Halfvarson J. Andersson A. F. Lucio M. Zheng Z. (2010). A Pyrosequencing Study in Twins Shows that Gastrointestinal Microbial Profiles Vary with Inflammatory Bowel Disease Phenotypes. Gastroenterology 139 (6), 1844–1854. e1841. 10.1053/j.gastro.2010.08.049 20816835

[B61] Wiśniewski J. R. Zougman A. Nagaraj N. Mann M. (2009). Universal Sample Preparation Method for Proteome Analysis. Nat. Methods 6 (5), 359–362. 10.1038/nmeth.1322 19377485

[B62] Yu N. Y. Wagner J. R. Laird M. R. Melli G. Rey S. Lo R. (2010). PSORTb 3.0: Improved Protein Subcellular Localization Prediction with Refined Localization Subcategories and Predictive Capabilities for All Prokaryotes. Bioinformatics 26 (13), 1608–1615. 10.1093/bioinformatics/btq249 20472543 PMC2887053

