# Supplementary material for: An Integrative Multiomics Approach to Characterize Prebiotic Inulin Effects on Faecalibacterium prausnitzii
Source: Front Bioeng Biotechnol. 2022 Jan 18;10:825399. doi: 10.3389/fbioe.2022.825399 (PMC8894670; doi:10.3389/fbioe.2022.825399)
Supplement: Supplementary file 2 [file DataSheet1.docx]

**Supplementary Table S1.** Proteomic changes of *F. prausnitzii* during growth in inulin, compared to growth in glucose. Proteomic analysis was performed in triplicates. *p*-values were provided using the Perseus software.

| **Protein name** | **Fold change (Log2)** | ***p*-value (-Log10)** | **UniProt ID** |
| --- | --- | --- | --- |
| Glycosyl hydrolase family 32 | 8.030 | 5.017 | C7H8W7 |
| Alpha amylase, catalytic domain protein | 8.024 | 5.135 | C7H8X5 |
| ABC transporter, permease protein | 6.727 | 2.877 | C7H8X0 |
| ABC transporter, permease protein | 6.429 | 4.095 | C7H8W9 |
| zf-HC2 domain-containing protein | 5.459 | 3.050 | C7H5A8 |
| Phosphoenolpyruvate-dependent sugar phosphotransferase system, EIIA 2 | 5.158 | 4.594 | C7H377 |
| ABC transporter, ATP-binding protein | 4.974 | 4.455 | C7H529 |
| Bacterial transferase hexapeptide repeat protein | 4.784 | 3.670 | C7H314 |
| DEAD/DEAH box helicase | 4.748 | 3.419 | C7H8I8 |
| ABC transporter, ATP-binding protein (Fragment) | 4.582 | 4.340 | C7H4V1 |
| Tagatose-6-phosphate kinase | 4.579 | 2.319 | C7H376 |
| 50S ribosomal protein L29 | 4.374 | 3.141 | C7H942 |
| Tat pathway signal sequence domain protein | 4.194 | 4.670 | C7H8W8 |
| Uncharacterized protein | 4.106 | 3.134 | C7H8X3 |
| Kinase, PfkB family | 3.958 | 4.748 | C7H8X4 |
| Carboxynorspermidine/carboxyspermidine decarboxylase | 3.864 | 1.371 | C7H603 |
| Transcriptional regulator, GntR family | 3.860 | 2.500 | C7H528 |
| Hsp20/alpha crystallin family protein | 3.730 | 4.193 | C7H7C9;C7H579 |
| Heat-inducible transcription repressor HrcA | 3.497 | 2.788 | C7H8G7 |
| Transcriptional repressor NrdR | 3.494 | 2.592 | C7H7U0 |
| 50S ribosomal protein L32 | 3.489 | 1.598 | C7H5Z1 |
| ABC transporter, ATP-binding protein (Fragment) | 3.445 | 2.936 | C7H371 |
| Dipeptidase | 3.401 | 2.254 | C7H311;C7HAZ3;C7HAZ4 |
| Uncharacterized protein | 3.354 | 1.765 | C7H876 |
| Hydrolase, alpha/beta domain protein | 3.343 | 1.687 | C7H8B9 |
| Putative Na/Pi-cotransporter II-like protein | 3.253 | 2.634 | C7H778 |
| Transcriptional regulator, DeoR family | 3.232 | 2.221 | C7H375 |
| Oxidoreductase, aldo/keto reductase family protein | 3.211 | 2.200 | C7H166 |
| Acetyltransferase, GNAT family | 3.149 | 1.394 | C7H1F6 |
| Arginine biosynthesis bifunctional protein ArgJ | 3.117 | 3.510 | C7H1M8 |
| 30S ribosomal protein S21 | 3.058 | 2.152 | C7H5V7 |
| 50S ribosomal protein L19 | 3.018 | 2.534 | C7H7V5 |
| GroES-like protein | 2.950 | 1.918 | C7H582 |
| Uncharacterized protein | 2.938 | 2.323 | C7H2J9 |
| Uncharacterized protein | 2.805 | 1.399 | C7H479 |
| ATP synthase subunit b | 2.794 | 1.348 | C7H675 |
| SCP-like protein | 2.751 | 2.507 | C7H4N7 |
| RmuC domain protein | 2.734 | 2.954 | C7H3S0 |
| Anaerobic ribonucleoside-triphosphate reductase-activating protein | 2.707 | 1.877 | C7H8Y0 |
| GTPase Obg | 2.701 | 1.855 | C7H7B9 |
| Transporter, major facilitator family protein | 2.668 | 1.832 | C7H434 |
| Uncharacterized protein | 2.651 | 2.227 | C7H9L7 |
| Dipeptidase PepV | 2.640 | 1.882 | C7H1A6 |
| ABC transporter, ATP-binding protein | 2.631 | 2.399 | C7H9K7 |
| Phosphoesterase | 2.589 | 1.631 | C7H1T9 |
| MATE efflux family protein | 2.556 | 2.470 | C7HAQ4 |
| Thymidine kinase | 2.555 | 3.663 | C7H966 |
| 30S ribosomal protein S12 | 2.523 | 1.882 | C7H3D1 |
| ABC transporter, ATP-binding protein | 2.486 | 1.602 | C7H2C0 |
| ABC transporter, ATP-binding protein | 2.467 | 2.301 | C7H6B3 |
| Exopolysaccharide biosynthesis polyprenyl glycosylphosphotransferase | 2.434 | 1.454 | C7H3X9 |
| Uncharacterized protein | 2.423 | 2.754 | C7H8K6 |
| Molybdopterin molybdenumtransferase | 2.415 | 1.507 | C7H4Q1 |
| AAA domain-containing protein | 2.398 | 2.558 | C7H281 |
| Precorrin-6A reductase | 2.393 | 1.491 | C7H9A8 |
| RNA methyltransferase, TrmH family | 2.292 | 3.149 | C7HA00 |
| Methylenetetrahydrofolate--tRNA-(uracil-5-)-methyltransferase TrmFO | 2.289 | 1.896 | C7H9Z7 |
| Antibiotic biosynthesis monooxygenase | 2.256 | 1.772 | C7H9U1 |
| Cysteine--tRNA ligase | 2.229 | 2.463 | C7H2Z3 |
| Transcriptional regulator, LacI family | 2.229 | 1.393 | C7H8X1 |
| 10 kDa chaperonin | 2.201 | 1.507 | C7H5B7 |
| Acetylglutamate kinase | 2.199 | 1.675 | C7H1M6 |
| Metallo-beta-lactamase domain protein | 2.188 | 2.799 | C7H4D3 |
| ABC transporter, ATP-binding protein | 2.138 | 1.391 | C7H3P9 |
| GTPase Era | 2.098 | 1.490 | C7H7P8 |
| HTH_16 domain-containing protein | 2.061 | 1.932 | C7H1E2 |
| 4Fe-4S binding domain protein | 2.037 | 1.484 | C7H683 |
| FAD dependent oxidoreductase | 2.037 | 1.670 | C7H191 |
| Glycosyl hydrolase family 3 N-terminal domain protein | -2.036 | 1.607 | C7H1M1 |
| Histidine kinase | -2.089 | 1.904 | C7H6U0;C7H6Q9 |
| Band_7_1 domain-containing protein (Fragment) | -2.096 | 1.642 | C7H9W6 |
| PASTA domain protein | -2.098 | 1.789 | C7H7B0 |
| Tat pathway signal sequence domain protein | -2.125 | 4.356 | C7H4C6 |
| PBP_domain domain-containing protein | -2.125 | 3.154 | C7H389 |
| Pyruvate kinase | -2.166 | 2.411 | C7H7D1 |
| Pyruvate kinase | -2.256 | 3.774 | C7H7D0 |
| Uncharacterized protein | -2.268 | 2.428 | C7H4I9 |
| Uncharacterized protein (Fragment) | -2.304 | 2.391 | C7H9W7 |
| 4-phosphoerythronate dehydrogenase | -2.350 | 2.275 | C7H518 |
| Stage 0 sporulation protein A homolog | -2.461 | 2.027 | C7H7I5 |
| Galactose-1-phosphate uridylyltransferase | -2.473 | 2.211 | C7H3B2 |
| Resolvase, N-terminal domain protein | -2.476 | 1.484 | C7H566 |
| Uncharacterized protein | -2.478 | 1.362 | C7H6D2 |
| Uncharacterized protein | -2.496 | 1.326 | C7H6N8 |
| Tat pathway signal sequence domain protein | -2.517 | 2.500 | C7H9W0 |
| Putative translation elongation factor G | -2.523 | 3.366 | C7H269 |
| Acetyltransferase, GNAT family | -2.576 | 1.653 | C7H9G8 |
| Ribosomal RNA small subunit methyltransferase A | -2.603 | 3.006 | C7H504 |
| Carbohydrate ABC transporter, carbohydrate-binding protein | -2.610 | 2.516 | C7H5Z8 |
| Peptidase, M23 family | -2.617 | 2.348 | C7H2L8 |
| Tat pathway signal sequence domain protein | -2.676 | 2.088 | C7H9T8 |
| Glycosyl hydrolase, family 31 | -2.708 | 2.279 | C7H817 |
| Ketopantoate reductase PanE/ApbA | -2.730 | 3.348 | C7H4L5 |
| Beta-galactosidase | -2.735 | 2.257 | C7H3E5 |
| Resolvase, N-terminal domain protein | -2.862 | 4.128 | C7H290 |
| ATP synthase epsilon chain | -2.872 | 1.369 | C7H349 |
| Uncharacterized protein | -2.884 | 2.217 | C7H1N9 |
| von Willebrand factor type A domain protein | -2.887 | 1.578 | C7H576 |
| Bro-N domain-containing protein | -2.957 | 2.449 | C7H9F3 |
| Transcriptional regulator, AsnC family | -3.048 | 1.818 | C7HAX8 |
| DUF4428 domain-containing protein | -3.154 | 1.676 | C7H9W8 |
| Uncharacterized protein | -3.160 | 3.216 | C7HAS4 |
| Mannitol dehydrogenase domain protein | -3.162 | 3.352 | C7H488 |
| Ricin-type beta-trefoil lectin domain protein | -3.177 | 1.561 | C7H8G0 |
| SAF domain protein | -3.192 | 2.916 | C7H487 |
| Toxin-antitoxin system, toxin component, RelE family | -3.216 | 2.474 | C7H336 |
| Uncharacterized protein | -3.235 | 2.034 | C7H335 |
| Ser/Thr phosphatase family protein | -3.249 | 3.515 | C7H9X6 |
| Uncharacterized protein | -3.254 | 2.439 | C7H563 |
| Uncharacterized protein | -3.345 | 1.936 | C7H9X0 |
| Oxidoreductase, NAD-binding domain protein | -3.385 | 1.757 | C7H7P2 |
| Uncharacterized protein | -3.509 | 2.320 | C7H1L9 |
| Uncharacterized protein | -3.546 | 3.487 | C7H1T0 |
| Uncharacterized protein | -3.625 | 3.235 | C7H5M9 |
| Bacterial transferase hexapeptide repeat protein | -3.728 | 3.044 | C7H1U1 |
| Uncharacterized protein | -3.728 | 1.910 | C7H9X3 |
| FtsK/SpoIIIE family protein | -3.793 | 1.756 | C7HAC8 |
| Uncharacterized protein | -3.799 | 2.335 | C7H6M4 |
| Flavodoxin | -3.940 | 4.370 | C7H5R9 |
| ATP synthase subunit a | -4.106 | 2.575 | C7H357 |
| DNA helicase | -4.183 | 3.230 | C7HAB6 |
| Uncharacterized protein | -4.278 | 2.928 | C7H564 |
| Putative phage tail component domain protein | -4.601 | 3.259 | C7H1J1 |
| DUF5046 domain-containing protein | -4.824 | 3.217 | C7H388 |
| AP endonuclease, family 2 | -4.867 | 3.270 | C7H7P4 |
| Uncharacterized protein | -5.173 | 3.752 | C7HA14 |
| Transporter, major facilitator family protein | -5.679 | 3.375 | C7H8V6 |
| Metallo-beta-lactamase domain protein | -5.723 | 3.523 | C7H836 |
| Enolase | -5.967 | 5.845 | C7H462 |
| Ferrous iron transport protein B | -6.093 | 1.510 | C7H443 |

**Supplementary Table S2.** List of top 10 BLAST hits (E-value <0.0001) using β-fructosidase as query in the UniProt database. The hits with the highest identity (%) are listed.

| **Protein name** | **Organism** | **Identity (%)** | **Gene name (ORF)** |
| --- | --- | --- | --- |
| Glycosyl hydrolase family 32 | *Faecalibacterium prausnitzii* (strain DSM 17677 / JCM 31915 / A2-165) | 100% | FAEPRAA2165_02761 |
| β-fructosidases (Levanase/invertase) (EC 3.2.1.26) | *Faecalibacterium prausnitzii* L2-6 | 95.7% | FP2_08710 |
| Sucrose-6-phosphate hydrolase (EC 3.2.1.26) | *Faecalibacterium prausnitzii* | 95.3% | sacA, ERS852426_01856 |
| β-fructofuranosidase | *Gemmiger formicilis* | 85.3% | SAMN02745178_01832 |
| Sucrose-6-phosphate hydrolase | *Faecalibacterium* sp. An121 | 68.8% | B5E66_12090 |
| β-fructofuranosidase | *Ruminococcaceae bacterium* D16 | 56.4% | HMPREF0866_00162 |
| Sucrose-6-phosphate hydrolase | *Flavonifractor* sp. An135 | 56.1% | B5E80_09340 |
| Sucrose-6-phosphate hydrolase (EC 3.2.1.26) | *Eubacterium plexicaudatum* ASF492 | 54.5% | C823_04082 |
| Sucrose-6-phosphate hydrolase (EC 3.2.1.26) | *Firmicutes bacterium* ASF500 | 54.4% | N510_01164 |
| Sucrose-6-phosphate hydrolase (EC 3.2.1.26) | *Eubacterium plexicaudatum* ASF492 | 54.0% | C823_00516 |

**Supplementary Table S3.** List of sequences producing significant alignments (E-value <0.0001) with β-fructosidase as query sequence among other *F. prausnitzii* strains. A NCBI BLASTP online tool was used for search.

| **Protein name** | ***F. praunsitzii* strain** | **Protein**  **Accession** | **Identity (%)** |
| --- | --- | --- | --- |
| glycosyl hydrolase family 32 | A2-165 | EEU95822 | 100 |
| sucrose-6-phosphate hydrolase | CNCM I 4543 | PDX66164 | 98.17 |
| sucrose-6-phosphate hydrolase | CNCM I 4574 | PDX78066 | 98.17 |
| glycoside hydrolase family 32 protein | MCC588 | MBT9714222 | 97.96 |
| glycoside hydrolase family 32 protein | MCC589 | MBT9690675 | 97.76 |
| glycoside hydrolase family 32 protein | BIOML-A1 | MSC63249 | 97.76 |
| sucrose-6-phosphate hydrolase | APC942/8-14-2 | RAW51895 | 97.35 |
| sucrose-6-phosphate hydrolase | CNCM I 4644 | PDX84322 | 97.35 |
| glycoside hydrolase family 32 protein | DFI.5.42 | MBV0898878 | 97.15 |
| sucrose-6-phosphate hydrolase | ATCC 27768 | RCH44724 | 97.15 |
| sucrose-6-phosphate hydrolase | ATCC 27766 | RCH49258 | 97.15 |
| glycoside hydrolase family 32 protein | AF10-13 | RGW77778 | 96.95 |
| sucrose-6-phosphate hydrolase | CNCM I 4544 | PDX70767 | 96.95 |
| sucrose-6-phosphate hydrolase | APC923/51-1 | RAW56698 | 96.95 |
| glycoside hydrolase family 32 protein | AF36-11AT | RGB70110 | 96.95 |
| sucrose-6-phosphate hydrolase | AHMP21 | PDX88766 | 96.95 |
| sucrose-6-phosphate hydrolase | APC923/61-1 | RAW58873 | 96.74 |
| glycoside hydrolase family 32 protein | AM36-18BH | RHC42788 | 96.54 |
| sucrose-6-phosphate hydrolase | CNCM I 4542 | PLK28850 | 96.54 |
| sucrose-6-phosphate hydrolase | Indica | ATL90320 | 96.54 |
| sucrose-6-phosphate hydrolase | 942/30-2 | AXA82442 | 96.54 |
| sucrose-6-phosphate hydrolase | APC942/18-1 | RAW49752 | 96.54 |
| glycosyl hydrolase family 32 | KLE1255 | EFQ05847 | 96.33 |
| sucrose-6-phosphate hydrolase | APC942/32-1 | RAW49618 | 96.33 |
| glycoside hydrolase family 32 protein | AM42-11AC | RGC07217 | 96.13 |
| glycoside hydrolase family 32 protein | AM39-7BH | RGC32863 | 96.13 |
| glycoside hydrolase family 32 protein | AF31-14AC | RGB85546 | 96.13 |
| sucrose-6-phosphate hydrolase | CNCM I 4644 | PDX83594 | 96.13 |
| glycoside hydrolase family 32 protein | AF29-11BH | RGB96069 | 95.93 |
| glycoside hydrolase family 32 protein | AM33-14AC | RGC37558 | 95.93 |
| sucrose-6-phosphate hydrolase | AHMP21 | PDX85631 | 95.72 |
| Beta-fructosidases (levanase/invertase) | L2/6 | CBK98452 | 95.72 |
| glycoside hydrolase family 32 protein | BIOML-B2 | MSC67007 | 95.72 |
| glycoside hydrolase family 32 protein | BIOML-B4 | MSC73072 | 95.72 |
| glycoside hydrolase family 32 protein | BIOML-B3 | MSC97965 | 95.72 |
| glycoside hydrolase family 32 protein | BIOML-B15 | MSD38720 | 95.72 |
| glycoside hydrolase family 32 protein | BIOML-B18 | MSD49915 | 95.72 |
| sucrose-6-phosphate hydrolase | APC922/41-1 | RAW61970 | 95.52 |
| glycoside hydrolase family 32 protein | DFI.5.40 | MBU8989818 | 95.52 |
| glycoside hydrolase family 32 protein | BIOML-B10 | MSC46244 | 95.52 |
| glycoside hydrolase family 32 protein | BIOML-B11 | MSC49893 | 95.52 |
| Sucrose-6-phosphate hydrolase | 2789STDY5608869 | CUN96358 | 95.32 |
| glycoside hydrolase family 32 protein | AF32-8AC | RGB91527 | 95.32 |
| sucrose-6-phosphate hydrolase | CNCM I 4546 | PDX72360 | 95.11 |
| sucrose-6-phosphate hydrolase | CNCM I 4575 | PDX80373 | 94.91 |
| Sucrose-6-phosphate hydrolase | 2789STDY5834970 | CUN12928 | 94.91 |
| glycoside hydrolase family 32 protein | AM37-13AC | RGC15447 | 94.7 |
| glycoside hydrolase family 32 protein | APC918/95b | AXB28040 | 94.5 |
| glycoside hydrolase family 32 protein | MCC585 | MBT9707138 | 94.5 |
| glycoside hydrolase family 32 protein | DFI.5.48 | MBV0927599 | 94.5 |
| sucrose-6-phosphate hydrolase | APC924/119 | RAW65408 | 94.5 |
| glycoside hydrolase family 32 protein | BIOML-B1 | MSC52404 | 94.3 |

**Supplementary Table S4.** List of top 10 BLAST hits (E-value <0.0001) using amylosucrase as query in the UniProt database. The hits with the highest identity (%) are listed.

| **Protein name** | **Organism** | **Identity (%)** | **Gene name (ORF)** |
| --- | --- | --- | --- |
| Alpha amylase, catalytic domain protein | *Faecalibacterium prausnitzii* (strain DSM 17677 / JCM 31915 / A2-165) | 100% | FAEPRAA2165_02769 |
| Amylosucrase | *Faecalibacterium prausnitzii* | 99.2% | CGS57_09730 |
| Amylosucrase | *Faecalibacterium prausnitzii* | 98.9% | DW855_13210 |
| Amylosucrase (EC 2.4.1.4) | *Faecalibacterium prausnitzii* | 98.9% | ams, FPPS064S07_02467 |
| Amylosucrase | *Faecalibacterium prausnitzii* | 98.9% | DWZ89_11100 |
| Amylosucrase | *Faecalibacterium prausnitzii* sp. AF27-11BH | 98.8% | DWY76_04015 |
| Amylosucrase | *Faecalibacterium prausnitzii* | 98.8% | CGS54_00310 |
| Amylosucrase (EC 2.4.1.4) | Uncultured *Faecalibacterium prausnitzii* sp. | 98.8% | SAMEA3545278_01463 |
| Alpha amylase, catalytic domain protein | *Faecalibacterium* cf*. prausnitzii* KLE1255 | 98.8% | HMPREF9436_02692 |
| Amylosucrase | *Faecalibacterium prausnitzii* | 98.7% | CRH10_08405 |

**Supplementary Table S5.** List of sequences producing significant alignments (E-value <0.0001) with amylosucrase as query sequence among other *F. prausnitzii* strains. A NCBI BLASTP online tool was used for search.

| **Protein name** | ***F. praunsitzii* strain** | **Protein**  **Accession** | **Identity (%)** |
| --- | --- | --- | --- |
| alpha amylase, catalytic domain protein | A2-165 | EEU95830 | 100 |
| amylosucrase | CNCM I 4543 | PDX66156 | 99.18 |
| amylosucrase | CNCM I 4574 | PDX78058 | 99.18 |
| amylosucrase | AM37-13AC | RGC15455 | 98.85 |
| amylosucrase | AM39-7BH | RGC32871 | 98.85 |
| amylosucrase | AF36-11AT | RGB70102 | 98.85 |
| amylosucrase | MCC589 | MBT9690667 | 98.69 |
| amylosucrase | Indica | ATL90312 | 98.69 |
| amylosucrase | BIOML-A1 | MSC63242 | 98.69 |
| amylosucrase | APC923/51-1 | RAW56690 | 98.69 |
| amylosucrase | CNCM I 4544 | PDX70759 | 98.68 |
| alpha amylase, catalytic domain protein | KLE1255 | EFQ05856 | 98.68 |
| amylosucrase | AHMP21 | PDX88751 | 98.52 |
| Amylosucrase | 2789STDY5608869 | CUN96621 | 98.52 |
| amylosucrase | APC942/8-14-2 | RAW51887 | 98.52 |
| amylosucrase | ATCC 27768 | RCH44732 | 98.52 |
| amylosucrase | ATCC 27766 | RCH49250 | 98.52 |
| amylosucrase | APC942/32-1 | RAW49610 | 98.52 |
| Amylosucrase | 2789STDY5834970 | CUN13100 | 98.52 |
| amylosucrase | BIOML-B2 | MSC67016 | 98.52 |
| amylosucrase | BIOML-B4 | MSC73081 | 98.52 |
| amylosucrase | BIOML-B3 | MSC97956 | 98.52 |
| amylosucrase | BIOML-B15 | MSD38729 | 98.52 |
| amylosucrase | BIOML-B18 | MSD49906 | 98.52 |
| amylosucrase | APC918/95b | AXB28048 | 98.36 |
| alpha-amylase family protein | DFI.5.48 | MBV0927590 | 98.36 |
| amylosucrase | BIOML-B1 | MSC52413 | 98.36 |
| amylosucrase | APC924/119 | RAW65416 | 98.36 |
| amylosucrase | APC923/61-1 | RAW58881 | 98.36 |
| amylosucrase | MCC588 | MBT9714214 | 98.36 |
| amylosucrase | APC922/41-1 | RAW61978 | 98.36 |
| amylosucrase | AF31-14AC | RGB85538 | 98.36 |
| amylosucrase | AHMP21 | PDX85639 | 98.19 |
| amylosucrase | DFI.5.42 | MBV0898886 | 98.19 |
| amylosucrase | CNCM I 4542 | PLK28839 | 98.19 |
| amylosucrase | CNCM I 4575 | PDX80381 | 98.03 |
| amylosucrase | BIOML-B10 | MSC46253 | 98.03 |
| amylosucrase | BIOML-B11 | MSC49884 | 98.03 |
| amylosucrase | BIOML-B7 | MSC69408 | 98.03 |
| amylosucrase | BIOML-B13 | MSC75919 | 98.03 |
| amylosucrase | BIOML-B9 | MSC81191 | 98.03 |
| amylosucrase | AM42-11AC | RGC07209 | 98.03 |
| Glycosidases | L2/6 | CBK98444 | 98.02 |
| amylosucrase | AF29-11BH | RGB96077 | 97.87 |
| amylosucrase | AF10-13 | RGW77770 | 97.87 |
| amylosucrase | AM33-14AC | RGC37566 | 97.52 |
| amylosucrase | AF32-8AC | RGB91535 | 97.37 |
| amylosucrase | 942/30-2 | AXA82450 | 97.21 |
| amylosucrase | APC942/18-1 | RAW49744 | 97.21 |
| amylosucrase | AM36-18BH | RHC42780 | 97.21 |
| amylosucrase | AF36-11AT | RGB67088 | 97.19 |
| alpha-amylase family protein | DFI.5.40 | MBU8989810 | 97.04 |
| amylosucrase | AHMP21 | PDX88411 | 97.03 |
| amylosucrase | AM42-11AC | RGC07044 | 95.71 |
| amylosucrase | AM39-7BH | RGC35352 | 95.71 |
| amylosucrase | CNCM I 4546 | PDX72352 | 93.55 |

**Supplementary Table S6.** Sequence of primers used for qRT-PCR.

| **Primer** | **Type** | **Primer sequence (5'-3')** | **Product length (bp)** |
| --- | --- | --- | --- |
| β-fructosidase | Forward | CTGCCACCGAGATGGATGAC | 200 |
|  | Reverse | CCGGAAGTCGAACCTGCTGA | 200 |
| Amylosucrase | Forward | GATGGCTGAAGCCTACAATG | 200 |
|  | Reverse | GCATCAGGTGCAGATAGGTC | 200 |
| GAPDH | Forward | CCAAGATGCTGGCTCACCTG | 200 |
|  | Reverse | GGTGCACTCCAGAACGACAT | 200 |

**Supplementary Table S7.** List of top 2 BLAST hits of (E-value <0.0001) the proteins upregulated in *F. prausnitzii* grown on inulin. The hits with the highest identity (%) are listed.

| ***Faecalibacterium prausnitzii* A2-165 protein** | |  | **BLAST hit protein** | |  |
| --- | --- | --- | --- | --- | --- |
| **Protein name** | **Predicted function** |  | **Protein name** | **Organism** | **Identity (%)** |
| Glycosyl hydrolase family 32 | β-fructosidase |  | β-fructosidases (Levanase/invertase) | *Faecalibacterium prausnitzii* L2-6 | 95.7% |
|  |  |  | Sucrose-6-phosphate hydrolase | *Faecalibacterium prausnitzii* | 95.3% |
| Alpha amylase, catalytic domain protein | Amylosucrase |  | Amylosucrase | *Faecalibacterium prausnitzii* | 99.2% |
|  |  |  | Amylosucrase | *Faecalibacterium prausnitzii* | 98.9% |
| ABC transporter, permease protein | Inulin-type fructan ABC transporter |  | Carbohydrate ABC transporter membrane protein 1, CUT1 family | *Faecalibacterium prausnitzii* L2-6 | 99.0% |
|  |  |  | ABC transporter permease | *Faecalibacterium prausnitzii* | 98.7% |
| ABC transporter, permease protein | Inulin-type fructan ABC transporter |  | Inner membrane ABC transporter permease protein ycjP | *Faecalibacterium prausnitzii* | 99.3% |
|  |  |  | Carbohydrate ABC transporter membrane protein 2, CUT1 family | *Faecalibacterium prausnitzii* L2-6 | 98.7% |
| Phosphoenolpyruvate-dependent sugar phosphotransferase system, EIIA 2 | Fructose PTS transporter |  | Phosphoenolpyruvate-dependent sugar phosphotransferase system EIIA 2 | *Faecalibacterium prausnitzii* sp. CAG:82 | 90.3% |
|  |  |  | PTS system, fructose-specific IIC component | *Gemmiger formicilis* | 88.1% |
| Tagatose-6-phosphate kinase | 1-phosphofructokinase |  | Tagatose-6-phosphate kinase | *Faecalibacterium prausnitzii* sp. CAG:82 | 86.9% |
|  |  |  | Tagatose-6-phosphate kinase | *Faecalibacterium prausnitzii* L2-6 | 85.7% |
| Tat pathway signal sequence domain protein | Inulin-type fructan ABC transporter extracellular solute-binding protein |  | Carbohydrate ABC transporter substrate-binding protein, CUT1 family | *Faecalibacterium prausnitzii* L2-6 | 97.5% |
|  |  |  | Lipoprotein lplA | *Faecalibacterium prausnitzii* | 96.2% |
| Uncharacterized protein | Uncharacterized protein |  | Uncharacterized protein | *Faecalibacterium prausnitzii* L2-6 | 99.0% |
|  |  |  | Uncharacterized protein | *Faecalibacterium prausnitzii* | 97.6% |
| Kinase, PfkB family | Fructokinase |  | Sugar kinases, ribokinase family | *Faecalibacterium prausnitzii* L2-6 | 97.4% |
|  |  |  | 5-dehydro-2-deoxygluconokinase | *Faecalibacterium prausnitzii* | 97.4% |
| Transcriptional regulator, DeoR family | Transcriptional regulator, DeoR family |  | DeoR/GlpR transcriptional regulator | *Faecalibacterium prausnitzii* | 91.6% |
|  |  |  | Transcriptional regulator DeoR family | Faecalibacterium prausnitzii sp. CAG:82 | 86.7% |
| Transcriptional regulator, LacI family | Transcriptional regulator, LacI family |  | Transcriptional regulators | *Faecalibacterium prausnitzii* L2-6 | 94.9% |
|  |  |  | Lactose operon repressor | *Faecalibacterium prausnitzii* | 92.9% |

**Supplementary Table S8.** Sequence alignment between proteins of *Faecalibacterium prausnitzii* A2-165 (UniProt Taxon No. is 411483) and *Roseburia Inulinivorans* A2-194 (UniProt Taxon No. is 622312).

| ***Faecalibacterium prausnitzii* A2-165** | ***Roseburia Inulinivorans* A2-194** | **Identity (%)** |
| --- | --- | --- |
| β-fructosidase | β-fructofuranosidase | 45.294% |
| Inulin-type fructan ABC transporter extracellular solute-binding protein | ABC sugar-binding protein | 55.232% |
| Inulin-type fructan ABC transporter | ABC sugar-binding protein | 45.981% |
| Inulin-type fructan ABC transporter | ABC sugar-binding protein | 66.667% |

**Supplementary Table S9.** Metabolomic changes of *F. prausnitzii* during growth in inulin, compared to growth in glucose. Metabolomic analysis was performed in quadruplicates. The FDR-corrected *p*-values were provided using the Metaboanalyst 5.0.

| **Metabolite name** | **Fold change (Log2)** | ***p-*value (-Log10)** |
| --- | --- | --- |
| Riboflavin | 4.21 | 3.62 |
| UDP-acetylglucosamine | 3.37 | 2.44 |
| Glyceric acid | 2.88 | 2.44 |
| Flavin mononucleotide | 1.89 | 2.51 |
| Erythrose phosphate | 1.7 | 2.91 |
| Deoxyuridine | 1.43 | 1.97 |
| Citrate | 1.23 | 3.34 |
| Galacturonic acid | 1.1 | 2.06 |
| Citrulline | 1.03 | 3.62 |
| Serotonin | 1 | 4.84 |
| Indoleacrylic acid | 1 | 2.53 |
| Palmitoyl-CoA | 0.99 | 1.76 |
| Aconitate | 0.98 | 1.50 |
| Tryptophan | 0.97 | 2.25 |
| Cytidine triphosphate | 0.94 | 1.95 |
| Fructose 6-phosphate | 0.92 | 1.50 |
| Succinyl-CoA | 0.92 | 1.40 |
| Glucose 6-phosphate | 0.89 | 1.50 |
| Cystathionine | 0.84 | 2.00 |
| Hydroxybutyric acid | 0.72 | 2.44 |
| Hydroxyectoine | 0.7 | 2.25 |
| Fructose 1,6-bisphosphate | -0.6 | 2.05 |
| Guanine | -0.65 | 1.48 |
| Adenosine triphosphate (ATP) | -0.66 | 3.23 |
| Valine | -0.66 | 2.25 |
| Lysine | -0.67 | 3.20 |
| Acetyl phosphate | -0.67 | 3.11 |
| Nicotinamide | -0.69 | 2.44 |
| Malonyl-CoA | -0.78 | 2.11 |
| Homoserine | -0.79 | 2.61 |
| Threonine | -0.79 | 2.61 |
| D-Biotin | -0.81 | 1.47 |
| Guanosine | -0.82 | 1.75 |
| Pyridoxamine | -0.84 | 2.02 |
| Aminobutyric acid | -0.85 | 2.51 |
| Deoxyinosine | -0.89 | 3.34 |
| Spermidine | -0.95 | 2.53 |
| Serine | -0.96 | 4.17 |
| glycine | -1 | 3.95 |
| Pyridinedicarboxylic acid | -1.03 | 2.44 |
| Deoxyadenosine monophosphate | -1.07 | 2.00 |
| Tyrosine | -1.17 | 3.87 |
| Asparate | -1.18 | 1.50 |
| Methionine sulfoxide | -1.19 | 1.71 |
| Homocysteine | -1.21 | 1.97 |
| Xanthosine monophosphate | -1.21 | 4.33 |
| Acetyl-CoA | -1.22 | 2.13 |
| Alanine | -1.29 | 3.62 |
| CoA | -1.3 | 1.72 |
| NAD | -1.31 | 3.62 |
| Deoxycytidine diphosphate | -1.36 | 2.61 |
| Xanthosine | -1.38 | 2.61 |
| Asparagine | -1.4 | 3.45 |
| Isoleucine | -1.47 | 3.31 |
| Proline | -1.5 | 4.84 |
| Orotate | -1.53 | 2.13 |
| Aminoadipic acid | -1.54 | 2.51 |
| Phenylalanine | -1.63 | 4.17 |
| Methionine | -1.66 | 5.42 |
| Deoxyadenosine triphosphate | -1.67 | 3.87 |
| Glutamate | -1.7 | 3.87 |
| Succinate | -1.76 | 3.08 |
| Cysteic acid | -1.81 | 3.41 |
| Niacin | -1.82 | 2.13 |
| Inosine | -1.87 | 2.13 |
| Malate | -1.92 | 3.62 |
| Glucose | -2.03 | 3.41 |
| Guanidineacetic acid | -2.06 | 4.84 |
| Aminovaleric acid | -2.19 | 3.86 |
| Fructose | -2.74 | 4.84 |
| UDP-glucuronic acid | -3.03 | 2.87 |
| Indole-carboxaldehyde | -3.53 | 3.04 |

**
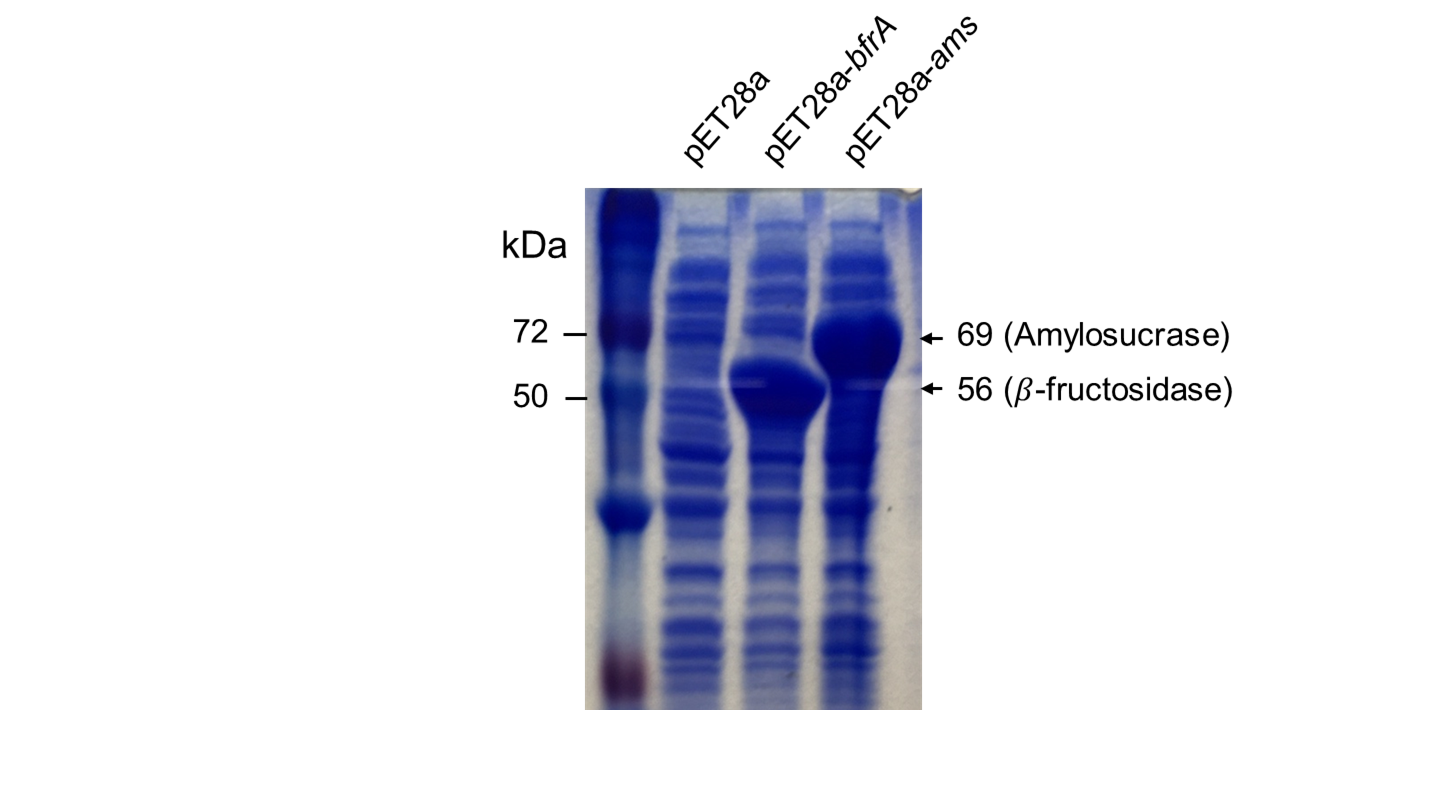
**

**Supplementary Figure 1.** The SDS–PAGE of recombinant strains. After cloning in pET28 and induction, the recombinant protein was analyzed by SDS-PAGE. SDS-PAGE showing expression of lysates of *E. coli* cells harboring pET28a, pET28a-*bfrA*, pET28a-*ams*.

**
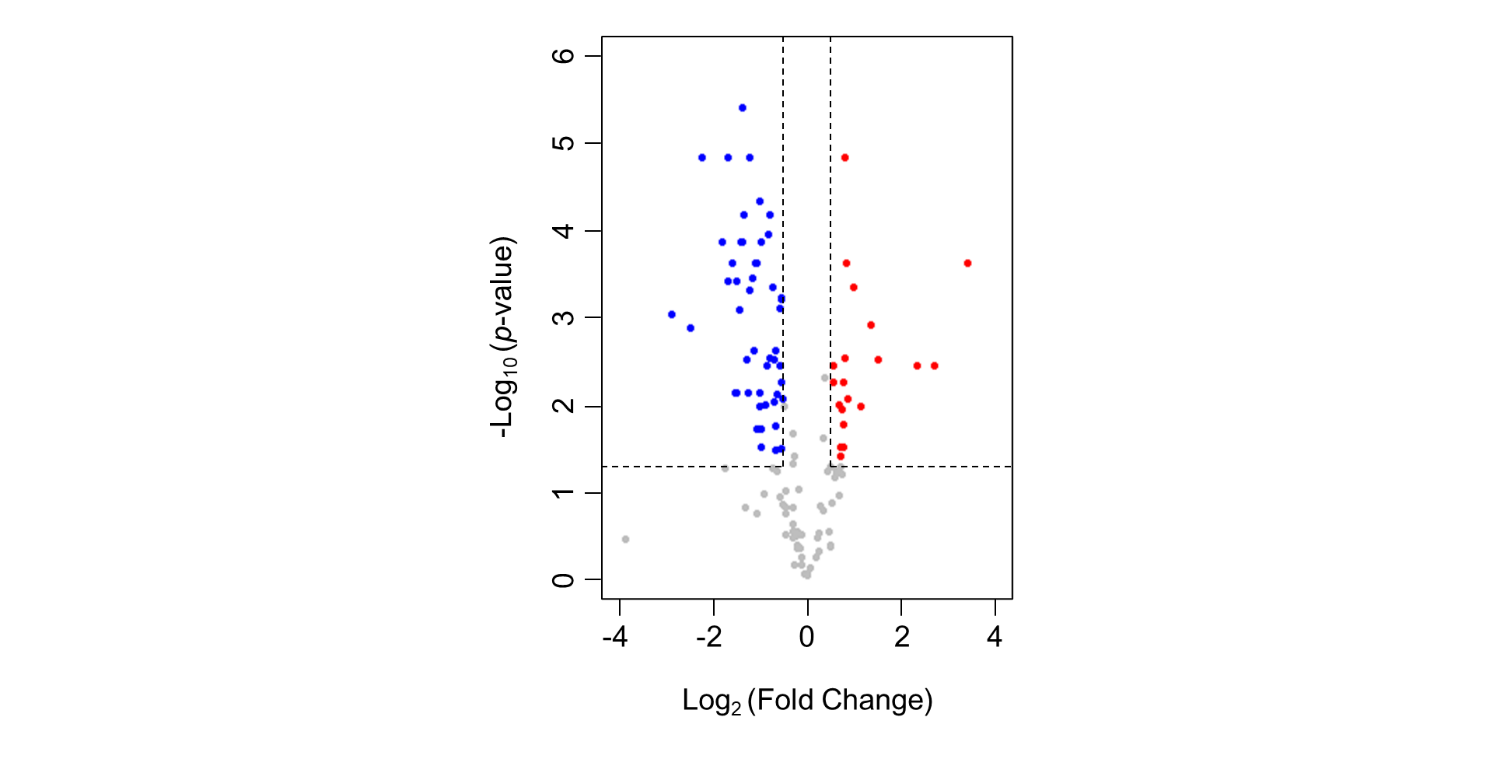
**

**Supplementary Figure 2.** Volcano plot of metabolomic analysis for *F. prausnitzii* grown in mYCFA supplemented with glucose or inulin. Red color indicates relative high expression in inulin-grown *F. prausnitzii* and blue color indicates relatively low expression in inulin-grown *F. prausnitzii*. Metabolomic analysis was performed in quadruplicates.
